# Increased carvone production in *Escherichia coli* by balancing limonene conversion enzyme expression via targeted quantification concatamer proteome analysis

**DOI:** 10.1038/s41598-021-01469-y

**Published:** 2021-11-11

**Authors:** Erika Yoshida, Motoki Kojima, Munenori Suzuki, Fumio Matsuda, Kazutaka Shimbo, Akiko Onuki, Yousuke Nishio, Yoshihiro Usuda, Akihiko Kondo, Jun Ishii

**Affiliations:** 1 Graduate School of Science, Technology and Innovation, Kobe University, 1-1 Rokkodai, Nada, Kobe, 657-8501 Japan; 2 Research Institute for Bioscience Products & Fine Chemicals. Ajinomoto Co, Inc. 1-1 Suzuki-cho, Kawasaki-ku, Kawasaki-shi, Kanagawa, 210-8681 Japan; 3 KNC Bio Research Center, KNC Laboratories Co, Ltd. 1-1-1, Murotani, Nishi-ku, Kobe, 651-2241 Japan; 4 Department of Bioinformatic Engineering, Graduate School of Information Science and Technology, Osaka University, 1-5 Yamadaoka, Suita, Osaka 565-0871 Japan; 5 Engineering Biology Research Center, Kobe University, 1-1 Rokkodai, Nada, Kobe, 657-8501 Japan; 6 Department of Chemical Science and Engineering, Graduate School of Engineering, Kobe University, 1-1 Rokkodai, Nada, Kobe, 657-8501 Japan

**Keywords:** Industrial microbiology, Metabolic engineering

## Abstract

(−)-Carvone is a monoterpenoid with a spearmint flavor. A sustainable biotechnological production process for (−)-carvone is desirable. Although all enzymes in (−)-carvone biosynthesis have been functionally expressed in *Escherichia coli* independently, the yield was low in previous studies. When cytochrome P450 limonene-6-hydroxylase (P450)/cytochrome P450 reductase (CPR) and carveol dehydrogenase (CDH) were expressed in a single strain, by-product formation (dihydrocarveol and dihydrocarvone) was detected. We hypothesized that P450 and CDH expression levels differ in *E. coli*. Thus, two strains independently expressing P450/CPR and CDH were mixed with different ratios, confirming increased carvone production and decreased by-product formation when CDH input was reduced. The optimum ratio of enzyme expression to maximize (−)-carvone production was determined using the proteome analysis quantification concatamer (QconCAT) method. Thereafter, a single strain expressing both P450/CPR and CDH was constructed to imitate the optimum expression ratio. The upgraded strain showed a 15-fold improvement compared to the initial strain, showing a 44 ± 6.3 mg/L (−)-carvone production from 100 mg/L (−)-limonene. Our study showed the usefulness of the QconCAT proteome analysis method for strain development in the industrial biotechnology field.

## Introduction

(−)-Carvone is a monoterpenoid and the key flavor compound of spearmint essential oil^1^. It is utilized for its flavor and fragrance in confectionery and oral care^2^. The annual production of (−)-carvone is approximately 3800 tons, the majority of which (approximately 2000 tons per year) is produced via chemical synthesis from (+)-limonene^3^. Owing to enhanced health- and environmental awareness in recent years, more consumers prefer natural flavors and fragrances^4^. Thus, the demand for natural spearmint flavor or natural (−)-carvone is increasing. Spearmint essential oil is currently the only natural spearmint flavor source, including natural (−)-carvone. The demand for natural spearmint flavor exceeds its supply. Increasing its supply is difficult as the current spearmint essential oil production method is water-intensive and requires improvement for sustainability. Additionally, spearmint cultivation is easily affected by weather (e.g., drought), such that the spearmint essential oil supply volume and unit price fluctuates^5^. Therefore, it is desirable to develop a sustainable and stable natural (−)-carvone production method to accommodate market demands.

**Figure 1:**
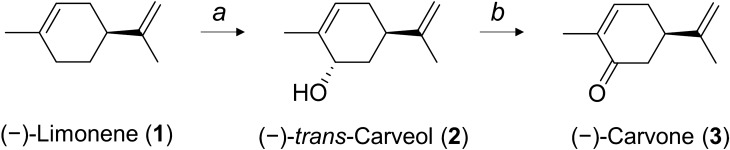
Biosynthesis pathway of carvone from limonene in *Mentha* sp*.* Biosynthesis pathway of (−)-carvone from the primary precursor (−)-limonene. The indicated enzymes are (**a**) cytochrome P450 limonene-6-hydroxylase and (**b**) carveol dehydrogenase.

A possible solution is (−)-carvone production by microbial fermentation. Numerous attempts have been made to produce flavor and fragrance compounds through biotechnology rather than extracting from natural sources, as it is a more sustainable and stable approach^6^. The regulatory circumstance also supports such attempts. For instance, the flavor and fragrance compounds produced through biotechnology (regardless of the microbial or enzymatic process) can be labeled “natural,” according to European regulation CE 1334/2008^7^. Therefore, (−)-carvone produced through the biotechnology method can be labeled as natural (−)-carvone and sustainably replace natural (−)-carvone conventionally produced by spearmint extraction. To the best of our knowledge, natural (−)-carvone has not yet been produced via biotechnological approaches.

(−)-Carvone is synthesized from the precursor (−)-limonene in its native producer, spearmint. Specifically, intracellular (−)-limonene is converted to (−)-*trans*-carveol by cytochrome P450 limonene-6-hydroxylase (together with cytochrome P450 reductase; CPR), while (−)-*trans*-carveol is converted to (−)-carvone by carveol dehydrogenase (CDH) (Fig. 1). The enzymology of (−)-carvone biosynthesis in spearmint has been studied in detail^8,9^. Cytochrome P450 limonene-6-hydroxylase and CDH have been functionally expressed in *Escherichia coli*^10,11^. However, when these three genes were expressed in a single *E. coli* strain, an extremely low level of (−)-carvone (up to 2 μM) was obtained from whole-cell biocatalysis with (−)-limonene supplementation^12^. Although the reason for the low conversion rate is still unclear, one general problem among heterologous expression of cytochrome P450 of plant origin is the difficulty to express it in a heterologous host like *E. coli*^13^. To increase target compound production in *E. coli*, careful tweaking at the protein level to balance P450 expression and other enzymatic pathways is necessary^14^. Heterologous expression of cytochrome P450 limonene-6-hydroxylase requires intensive N-terminal modification^10^, whereas CDH was expressed in a soluble form without modification^11^. Kinetic parameter information of these two enzymes was limited, but it appeared that the P450 reaction was rate-limiting in spearmint plants^9^. Based on these prior studies, we hypothesized that the expression levels of P450 and CDH are quite different, leading to an imbalance in the carvone biosynthesis pathway in *E. coli*, ultimately resulting in a low conversion rate from (−)-limonene to (−)-carvone. To investigate this hypothesis, a protein quantification method with high sensitivity was required to conduct a comparative study among various strains by the abundance ratio of pathway enzymes.

Proteome analysis is a powerful tool for protein quantification. Proteome analysis can be divided into two types: relative and absolute quantification. The relative quantification method can be conducted without laborious preparations of standard proteins; however, the expression levels of different proteins cannot be compared. The absolute quantification method requires a synthetic, isotope-labeled standard peptide preparation^15^. Absolute quantification (AQUA) peptide preparation is still relatively expensive, thus, rarely performed in strain construction for industrial biotechnology applications. Another method is individual purification of target proteins in an isotope-labeled form, known as the protein standard absolute quantification (PSAQ) method^16^. Alternatively, the quantification concatamer (QconCAT) method makes standard peptide preparation easier and cheaper^17^. In the QconCAT method, targeted peptides are concatenated into a QconCAT protein, spiked as a proteome analysis standard. A few studies have used the QconCAT method for protein quantification in prokaryotes^18–20^; however, to the best of our knowledge, no previous study applied the QconCAT method to genetic engineering to upgrade metabolic pathways. In recent years, DNA synthesis has become extremely accessible, such that the QconCAT method has the potential to become more popular in synthetic biology.

Here, we developed a QconCAT method to quantify the expression of carvone biosynthetic pathway enzymes (P450, CPR, and CDH) and to develop a sustainable and cost-effective replacement of carvone production using microbes. To the best of our knowledge, this is the first report of genetic engineering to upgrade metabolic pathways using the QconCAT method.

## Results

### Cloning and functional expression of P450 genes in *E. coli*

**Figure 2:**
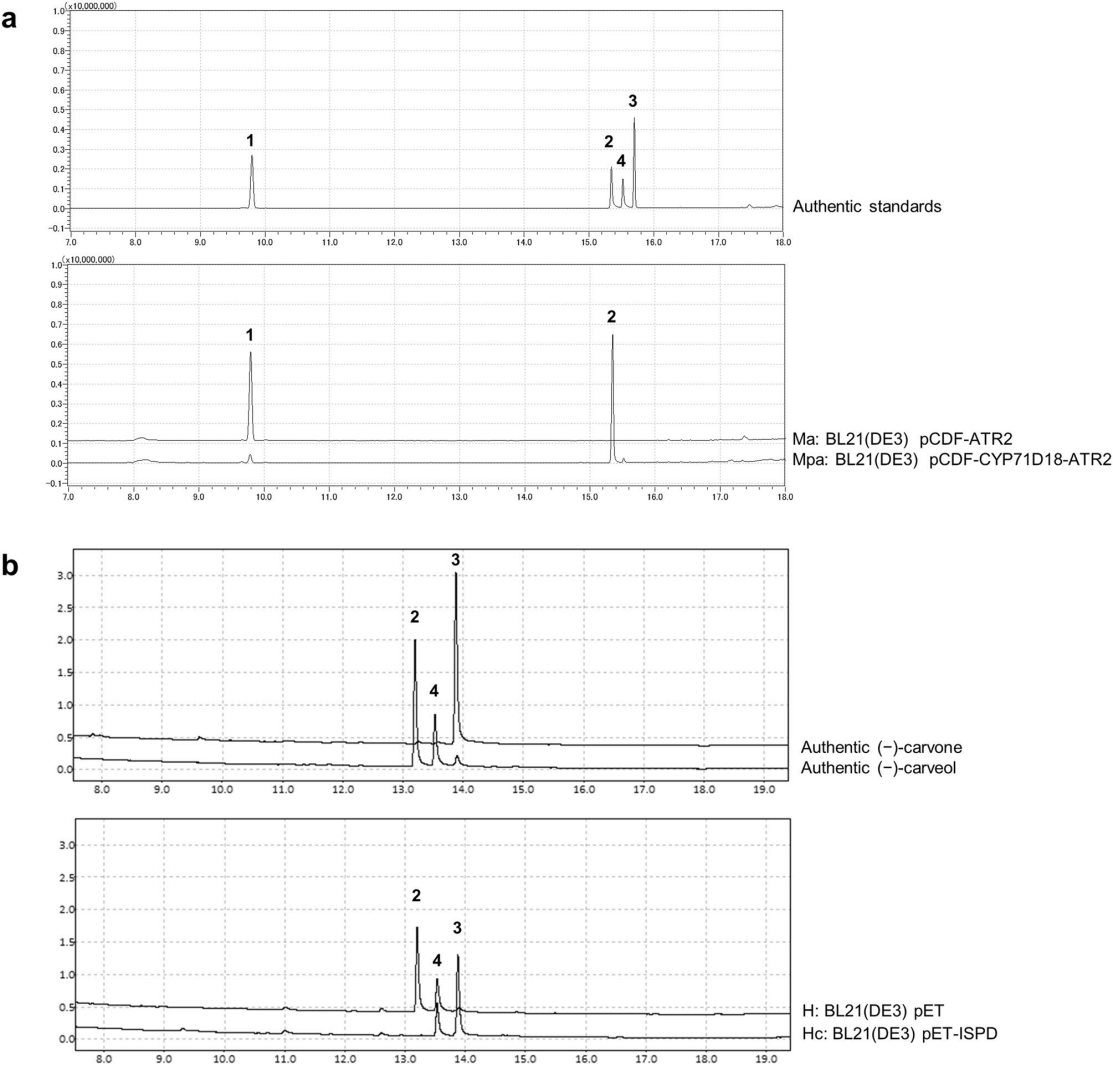
Limonene and carveol conversions for tests of constructed recombinant *Escherichia coli* strains using a whole-cell biocatalytic system. **(a)** Reaction with (–)-limonene and cells expressing the cytochrome P450 limonene-6-hydroxylase gene from spearmint (*CYP71D18*) and/or cytochrome P450 reductase (CPR) gene from *Arabidopsis thaliana* (*ATR2*) (Mpa and Ma strains; BL21(DE3) pCDF-CYP71D18-ATR2 and BL21(DE3) pCDF-ATR2)*.* Mpa strain specifically converted (−)-limonene to (−)-*trans*-carveol. **(b)** Reaction with (−)-carveol and cells expressing the carveol dehydrogenase (CDH) gene from peppermint (*ISPD*) (Hc strain; BL21(DE3) pET-ISPD). Hc strain specifically converted (−)-*trans*-carveol to (−)-carvone. Enzymatic activities were confirmed by gas chromatography (GC) analysis. Upper and lower panels indicate authentic standards and test samples; (−)-limonene (**1**), (−)-carveol (**2**, **4**), and (−)-carvone (**3**). Commercially available (−)-carveol contains (−)-*trans*-carveol (**2**), (−)-*cis*-carveol (**4**), and traces of (−)-carvone (**3**).

The P450 spearmint (*Mentha spicata*) gene, *CYP71D18,* was codon-optimized for *E. coli*, and the CPR gene *ATR2* of *Arabidopsis thaliana* was used as the native sequence. These genes were cloned into the pCDFDuet-1 vector and validated to be consistent with designed sequences. *E. coli* BL21(DE3) transformants harboring pCDF-ATR2 (named as Ma strain) or pCDF-CYP71D18-ATR2 (named as Mpa strain) were induced with isopropyl β-d-1-thiogalactopyranoside (IPTG), and whole-cell P450 enzymatic activity was tested with (−)-limonene supplementation as a substrate. The *E. coli* strain Mpa, which expresses both P450 and CPR, demonstrated the conversion of (−)-limonene to (−)-*trans*-carveol (Fig. 2a). The whole-cell activity was defined as the final concentration of (−)-*trans*-carveol per h per optical density of the culture at 600 nm (OD_600_). Mpa strain showed whole-cell P450 activity of 2.4 (mg × L^−1^ h^−1^ OD_600_^–1^).

### Cloning and functional expression of CDH genes in *E. coli*

Two genes were tested for CDH, one from peppermint (*Mentha* × *piperita*) and another from *Rhodococcus erythropolis*. First, the peppermint CDH gene, *ISPD*, was codon-optimized and cloned into the pET-3a vector. The sequence was validated to be consistent with the designed sequence. *E. coli* BL21(DE3) transformants harboring pET-ISPD (named as Hc strain) was induced with IPTG. Whole-cell CDH enzymatic activity was tested with (−)-carveol supplementation as a substrate (commercially available (−)-carveol contains (−)-*trans*-carveol and (−)-*cis*-carveol, and trace of (−)-carvone). The *E. coli* strain Hc, which expresses ISPD, demonstrated conversion of (−)-*trans*-carveol to (−)-carvone (Fig. 2b). CDH activity of ISPD-expressing cells was specific to (−)-*trans*-carveol and not toward (−)-*cis*-carveol, which is consistent with a previous study^11^. Whole-cell CDH activity was defined as final concentration (−)-carvone per h per OD_600_. Hc strain showed whole-cell CDH activity of 4.9 (mg × L^−1^ h^−1^ OD_600_^–1^).

In addition to CDH from peppermint (ISPD), another CDH gene, *limC* from *R. erythropolis* DCL14, was codon-optimized and cloned into pET-3a and expressed in *E. coli*. There were no previous reports of the heterologous expression of this gene. *E. coli* BL21(DE3) transformants harboring pET-limC were induced with IPTG under various conditions, and sodium dodecyl sulfate–polyacrylamide gel electrophoresis (SDS-PAGE) of the cell lysate revealed an evident band with a molecular mass of 30 kDa, corresponding to the size of CDH from *R. erythropolis* DCL14 (see Supplementary Fig. S1 online). This indicated that the CDH gene, *limC*, was successfully expressed in *E. coli* BL21(DE3). Thereafter, the CDH enzymatic activity of expressed LimC was tested with (−)-carveol supplementation as a substrate. The *E. coli* strain expressing the *limC* gene demonstrated (−)-carveol conversion to (−)-carvone only in the presence of the artificial electron acceptor dichlorophenolindophenol, as described in a previous study^21^ (see Supplementary Fig. S1 online). Additional supplementation of artificial electron acceptors is not desirable in industrial production; therefore, we selected the CDH gene, *ISPD*, from peppermint for the remaining study.

### Biocatalysis of (−)-carvone from (−)-limonene

**Figure 3:**
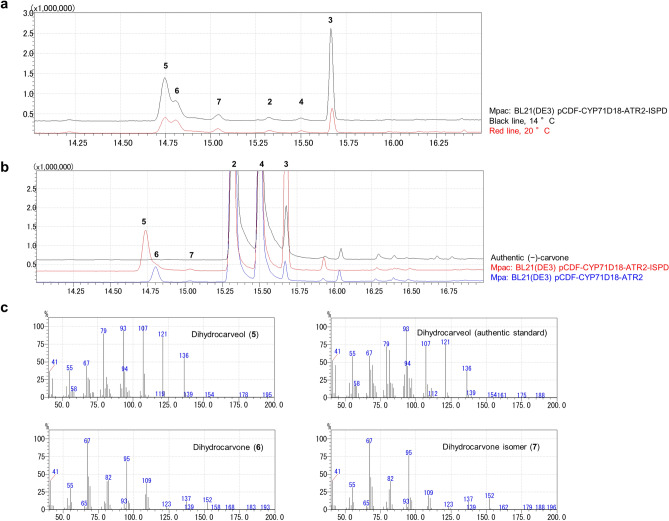
Carvone production from limonene using a recombinant *Escherichia coli* strain co-expressing P450, CPR, and CDH, and identification of by-product compounds. Cytochrome P450 limonene-6-hydroxylase gene from spearmint (*CYP71D18*), cytochrome P450 reductase (CPR) gene from *Arabidopsis thaliana* (*ATR2*) and carveol dehydrogenase (CDH) gene from peppermint (*ISPD*) were used as P450, CPR, and CDH, respectively. Their enzymatic activities were confirmed by gas chromatography (GC) analysis. (**a**) Reaction with (−)-limonene and cells co-expressing P450, CPR, and CDH (Mpac strain; BL21(DE3) pCDF-CYP71D18-ATR2-ISPD) under different temperature conditions (black line, 14 °C; and red line, 20 °C). Mpac strain converted (−)-limonene to (−)-carvone, and also generated by-products. (**b**) Reaction with (−)-carveol and cells expressing P450, CPR, and CDH (Mpac strain, red line), and cells expressing P450 and CPR (Mpa strain, blue line). The Mpac strain generated an undetermined by-product, compound (5). The Mpa strain generated an undetermined by-product, compound (6, 7). The black line indicates authentic (−)-carveol standard. Commercially available (−)-carveol contains (−)-*trans*-carveol (2), (−)-*cis*-carveol (4), and trace of (−)-carvone (3). (**c**) Mass spectrometry (MS) fragment pattern of reaction products with (−)-limonene and cells expressing P450, CPR and CDH (Mpac strain), and the authentic standard (dihydrocarveol). Fragment patterns determined by GC–MS analysis indicated that the undetermined by-products were dihydrocarveol and dihydrocarvone. Numbers indicate the following compounds: (−)-*trans*-carveol (2), (−)-carvone (3), (−)-*cis*-carveol (4), dihydrocarveol (5), dihydrocarvone (6), dihydrocarvone isomer (7).

The P450 spearmint gene, *CYP71D18*, the CPR gene *ATR2* of *A. thaliana*, and the CDH peppermint gene, ISPD, were individually confirmed to be functionally expressed in *E. coli*. Thereafter, a strain co-expressing P450, CPR, and CDH was constructed (BL21(DE3) pCDF-CYP71D18-ATR2-ISPD, named the Mpac strain) and incubated with (−)-limonene as a substrate at two different temperatures. Reaction temperatures were selected based on the preliminary study to determine the optimal reaction temperature of CYP71D18 expressing cells (see Supplementary Fig. S2 online). As a result, (−)-carvone was detected along with undesired by-product formation (Fig. 3a). Out of the two reaction temperature conditions tested, 14 °C showed higher (−)-carvone production than 20 °C; thus, we selected 14 °C as the reaction temperature for the remaining study. To identify the reason for by-product formation, the P450, CPR, and CDH co-expressing strain (Mpac), or P450/CPR expressing strain (Mpa) were incubated with (−)-carveol as a substrate, and different patterns of by-product formation were observed (Fig. 3b). Specifically, the Mpac strain expressing CDH generated compound #5, while the Mpa strain that did not express CDH generated compound #6 with a small quantity of compound #7 (Fig. 3b). A similarity search of the mass spectrometry (MS) fragment pattern and further analysis of the conversion mixture and authentic compound suggested that these by-products were dihydrocarveol (#5) and dihydrocarvone (#6) (Fig. 3c). The minor peak, #7, was the dihydrocarvone isomer (Fig. 3c). Besides, when (−)-carveol and (−)-carvone were incubated with the wild type *E. coli* BL21(DE3) strain, dihydrocarvone formation was also observed (see Supplementary Fig. S3 online). These results indicated that the exogenous peppermint enzyme CDH (ISPD) generated dihydrocarveol, and the endogenous *E. coli* enzyme generated dihydrocarvone as by-products.

### Optimization of carvone biocatalysis by QconCAT proteome analysis

**Figure 4:**
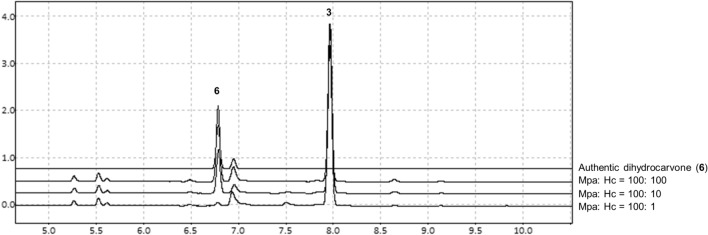
Optimization of enzyme balance by mixing two strains expressing P450/CPR and CDH enzymes. Strains expressing P450 and CPR (Mpa strain; BL21(DE3) pCDF-CYP71D18-ATR2) or CDH (Hc strain; BL21(DE3) pET-ISPD) were mixed in various ratios to determine the optimum balance for converting (−)-limonene to (−)-carvone. Varied P450/CDH input results in different (−)-carvone (3) production together with by-product dihydrocarvone (6) production.

CDH was shown to cause by-product formation; thus, optimizing the expression ratio between P450/CPR and CDH was attempted. This experiment was conducted under the hypothesis that excessive CDH in the reaction may cause undesired by-product formation. The Mpa strain (BL21(DE3) harboring pCDF-CYP71D18-ATR2) and Hc strain (BL21(DE3) harboring pET-ISPD) were separately cultured, induced with IPTG, and mixed at various ratios with (−)-limonene substrate. The results of Mpa and Hc strains mixed at 100:100, 100:10, and 100:1, based on the OD_600_ value, are shown in Fig. 4. When P450/CPR- and CDH-expressing strains were mixed at a ratio of 100:1, (−)-carvone concentration reached a maximum, while the dihydrocarvone peak was at a minimum. To corroborate this hypothesis, BL21(DE3) transformants harboring the high-copy CDH expression plasmid (pET-ISPD) along with pCDF-CYP71D18-ATR2 were generated (MpaHc strain) and confirmed that MpaHc, presumably with high CDH expression, drastically decreased carvone production. This result was also reproduced in a similar reaction condition (see Supplementary Fig. S4 online).

Thereafter, we aimed to determine the optimum ratio between P450/CPR and CDH at the protein level. One problem with this strategy is no method is available for protein quantification, especially P450. Heterologous expression of CYP71D18 was not detectable by SDS-PAGE analysis, while other proteins, such as CPR and CDH, were readily detectable. As shown in Fig. 4, reducing the amount of CDH in the reaction is beneficial for increasing (−)-carvone conversion. It is necessary to obtain the quantitative result of the protein expression level to determine the optimum abundance ratio among enzymatic pathways to be represented in the optimized strain. Therefore, a quantitative proteome analysis method using the QconCAT protein was developed for P450, CPR, and CDH quantification.

**Figure 5:**
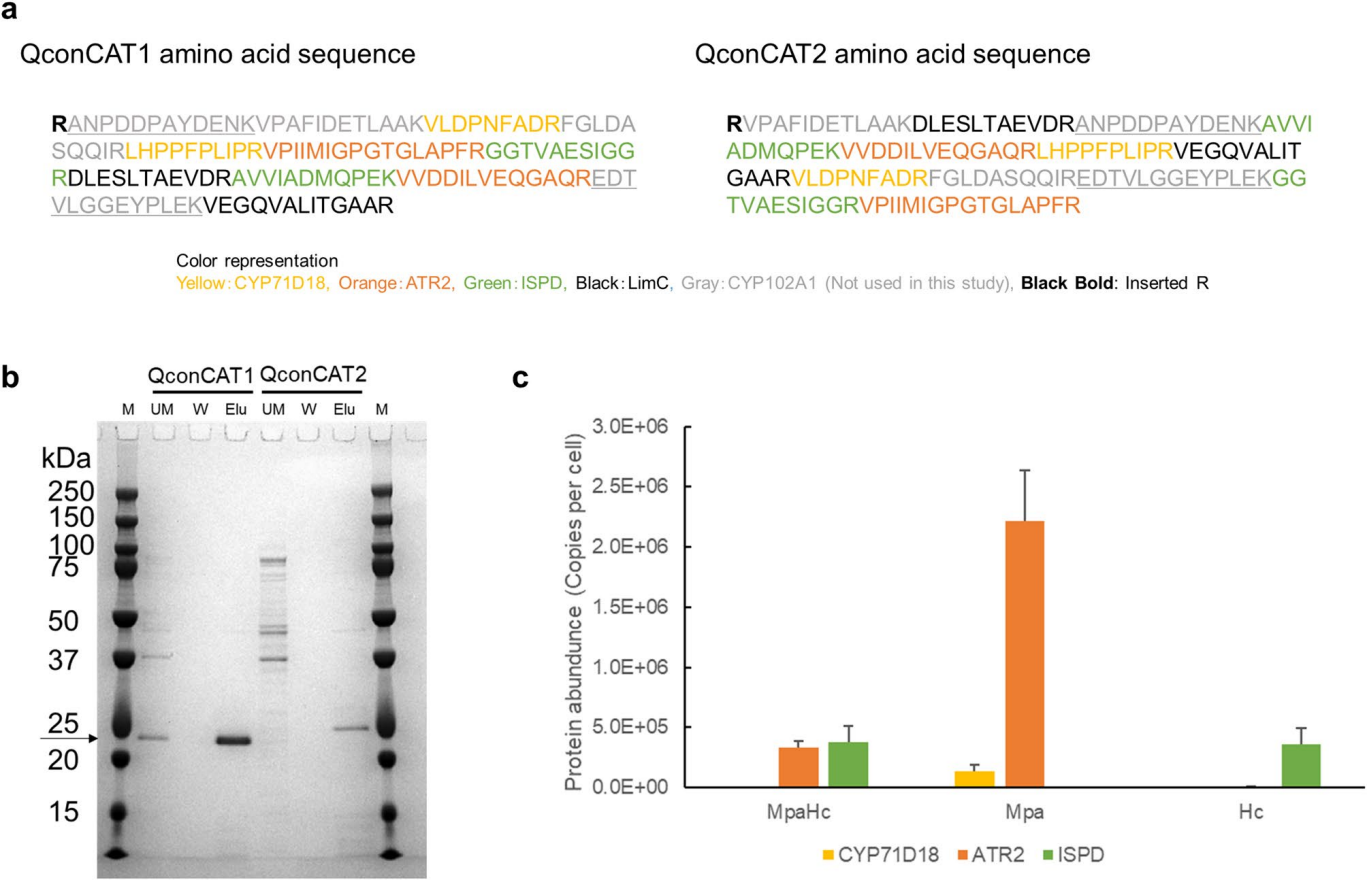
Quantification of enzyme expression level by proteome analysis using the quantification concatamer (QconCAT) method. To determine the optimum balance for converting limonene to carvone, quantitative proteome analysis using the QconCAT protein was conducted for strains expressing P450 (CYP71D18), CPR (ATR2), or CDH (ISPD). (**a**) QconCAT standard protein design. Two tryptic peptides were selected based on the preliminary study to represent each protein. The peptide sequences were concatenated in two different orders to produce QconCAT1 and QconCAT2. (**b**) Purification of QconCAT proteins. Sodium dodecyl sulfate–polyacrylamide gel electrophoresis (SDS-PAGE) image shows that QconCAT proteins (20 kDa, indicated by an arrow) were correctly expressed and purified. Applied samples per lane were indicated as follows; M: molecular mass standard marker, UM: material not bound to the his-tag column, W: wash fractions, Elu: elution fractions. Positions of molecular mass standards are indicated. (**c**) Quantitative proteome analysis using the QconCAT method. Unlabeled soluble proteins from strains expressing P450 (CYP71D18), CPR (ATR2), or CDH (ISPD) were mixed with labeled QconCAT, digested with trypsin and analyzed by nano-LC–MS/MS. The intensities of unlabeled and labeled peptides were used to calculate the absolute amounts of each protein, as copies of each protein per cell. The MpaHc strain (BL21(DE3) pCDF-CYP71D18-ATR2, pET-ISPD) expressed P450 (CYP71D18), CPR (ATR2), and CDH (ISPD). The Mpa strain (BL21(DE3) pCDF-CYP71D18-ATR2) expressed P450 (CYP71D18) and CPR (ATR2). The Hc strain (BL21(DE3) pET-ISPD) expressed CDH (ISPD). The protein abundance was calculated as an average of three independent clones. Error bars represent standard deviations of *n* = 3.

We first determined the candidate peptides to be analyzed based on the amino acid sequences of P450, CPR, and CDH. The peptide sequences from CYP102A1 (P450) and LimC (CDH) were included as well as those from CYP71D18 (P450), ATR2 (CPR) and ISPD (CDH) in QconCAT designs but were not used in quantification. As described above (Supplementary Fig. S1 online), LimC was not used due to the requirement for artificial electron acceptor supplementation. In contrast, CYP102A1 had been an alternative P450 candidate of CYP71D18, while we did not choose it for (−)-carvone bioconversion eventually in this study due to the low regioselectivity (data not shown). We analyzed actual samples from strains expressing these three proteins (CYP71D18, ATR2 and ISPD) and selected two peptides for each protein based on their detection strength. Artificial standard QconCAT proteins were subsequently constructed. In the QconCAT protein design, all peptides to be analyzed were sequentially concatenated. Two different designs were attempted by arranging peptides in different orders, namely, QconCAT1 and QconCAT2 (Fig. 5a). DNA sequences corresponding to these artificial proteins were synthesized and inserted into the pET-28a vector. *E. coli* BL21(DE3) transformants harboring pET28a-QconCAT1 or pET28a-QconCAT2 were induced with IPTG, and SDS-PAGE of the cell lysate revealed a band with a molecular mass of 20 kDa, which corresponds to the QconCAT protein as designed. Both were His-tagged purified to single-band purity (Fig. 5b).

These QconCAT proteins were treated with trypsin, and the detection of all peptides included in the design was confirmed in advance. We used the QconCAT1 protein, in which the yield after purification was higher than that after QconCAT2 (Fig. 5b), for further experiments. Thereafter, the strain expressing the QconCAT1 protein was cultured in a labeled medium in which glucose was substituted with [U-^13^C_6_] glucose, induced with IPTG, and purified as previously described. This labeled purified QconCAT1 protein was used as a standard for proteomic analysis.

To confirm the complete trypsin digestion, the mixture of unlabeled lysate and labeled QconCAT1 were analyzed in time course (see Supplementary Fig. S5 online). The trypsin digestion time was selected as 16 h of incubation. The test for miscleavage peptides at 16 h of trypsin digestion revealed the presence of miscleavage peptide VVDDILVEQGAQREDTVLGGEYPLEK (data not shown). Therefore, the second peptide derived from ATR2, VVDDILVEQGAQR was excluded from the quantification. The later peptide of the miscleaved peptide, EDTVLGGEYPLEK is derived from CYP102A1, which was not used in this study. The isotopomer profile of labeled QconCAT via data-dependent acquisition mode LC–MS/MS analysis revealed the presence of labeling deficit peaks with smaller *m/z* (CYP71D18 first peptide VLDPNFADR are shown as an example in Supplementary Fig. S6 online). Since the purity of [U-^13^C_6_] glucose was 99%, a labeling deficit caused by 1% impurity of [U-^13^C_6_] glucose is possible. The isotopomer profile of the unlabeled lysate sample showed a good agreement with calculated theoretical mass spectra (see Supplementary Fig. S7 online). The error caused by the QconCAT labeling deficit was shown to be small (see Supplementary Fig. S8 online), and to compensate for the labeling deficit effect, the quantification was conducted by subtracting the negative control value. The linearity of the QconCAT1 protein was assayed by measuring an external calibration curve for each peptide. For individual peptides, their calibration curves showed good linearity across a wide range of concentrations from 0.25 pmol to 2.5 nmol (see Supplementary Fig. S9 online). For CYP71D18 and ISPD, the correlation between values obtained with each peptide of the pair was shown in Supplementary Fig. S10 online, and the absolute quantification was calculated as an average of two peptides.

**Figure 6:**
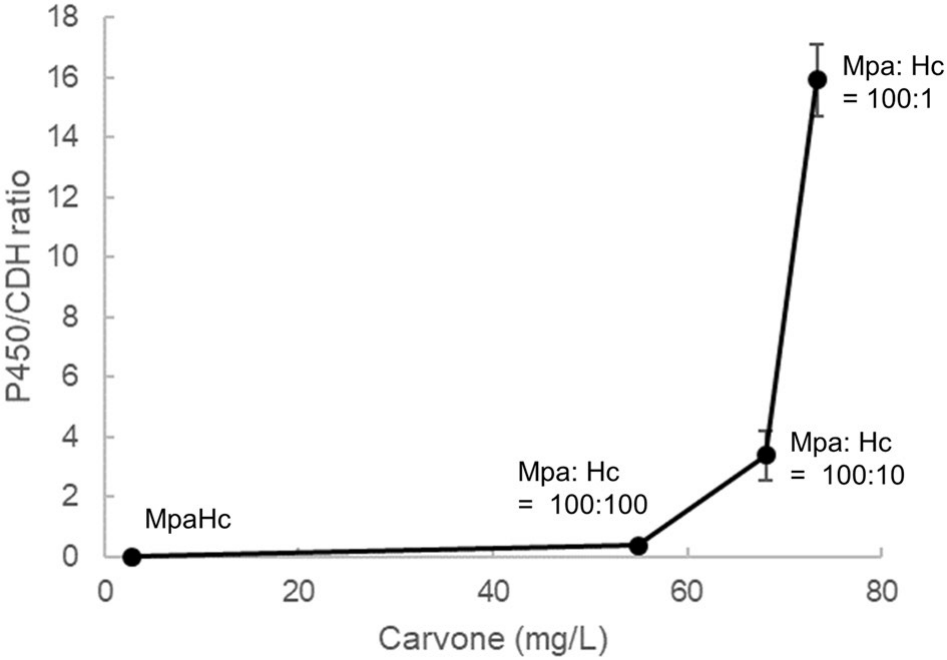
Correlation of the P450/CDH protein ratio and the amount of carvone produced. The P450/CDH ratio was calculated based on quantification concatamer (QconCAT) proteome analysis, and its correlation with carvone production is shown. Each plot on the graph represents four different conditions. From left to right: MpaHc (BL21(DE3) pCDF-CYP71D18-ATR2, pET-ISPD) showing a P450/CDH ratio of 0.004, Mpa (BL21(DE3) pCDF-CYP71D18-ATR2) and Hc (BL21(DE3) pET-ISPD) mixture (100:100) showing a P450/CDH ratio of 0.388, Mpa and Hc mixture (100:10) showing a P450/CDH ratio of 3.4, and Mpa and Hc mixture (100:1) showing a P450/CDH ratio of 16. The Mpa and Hc mixture ratio is based on the OD_**600**_ value of each strain.

The absolute protein abundance was determined by QconCAT method using strains expressing P450, CPR, and CDH (MpaHc), P450 and CPR (Mpa), or CDH (Hc) (Fig. 5c). It was shown that P450 (CYP71D18) expression was extremely low in the MpaHc strain, whereas CPR (ATR2) and CDH (ISPD) expression was high. Based on the QconCAT proteome analysis results, the P450/CDH ratio corresponding to the Mpa and Hc mixture (100:100, 100:10, 100:1 ratio based on the OD_600_ value) and MpaHc was calculated, and the correlation between carvone production was shown (Fig. 6). Under the tested conditions, (−)-carvone concentration reached a maximum when the P450/CDH ratio was 16 (Mpa:Hc = 100:1).

### Carvone conversion by single strain reaction

**Figure 7:**
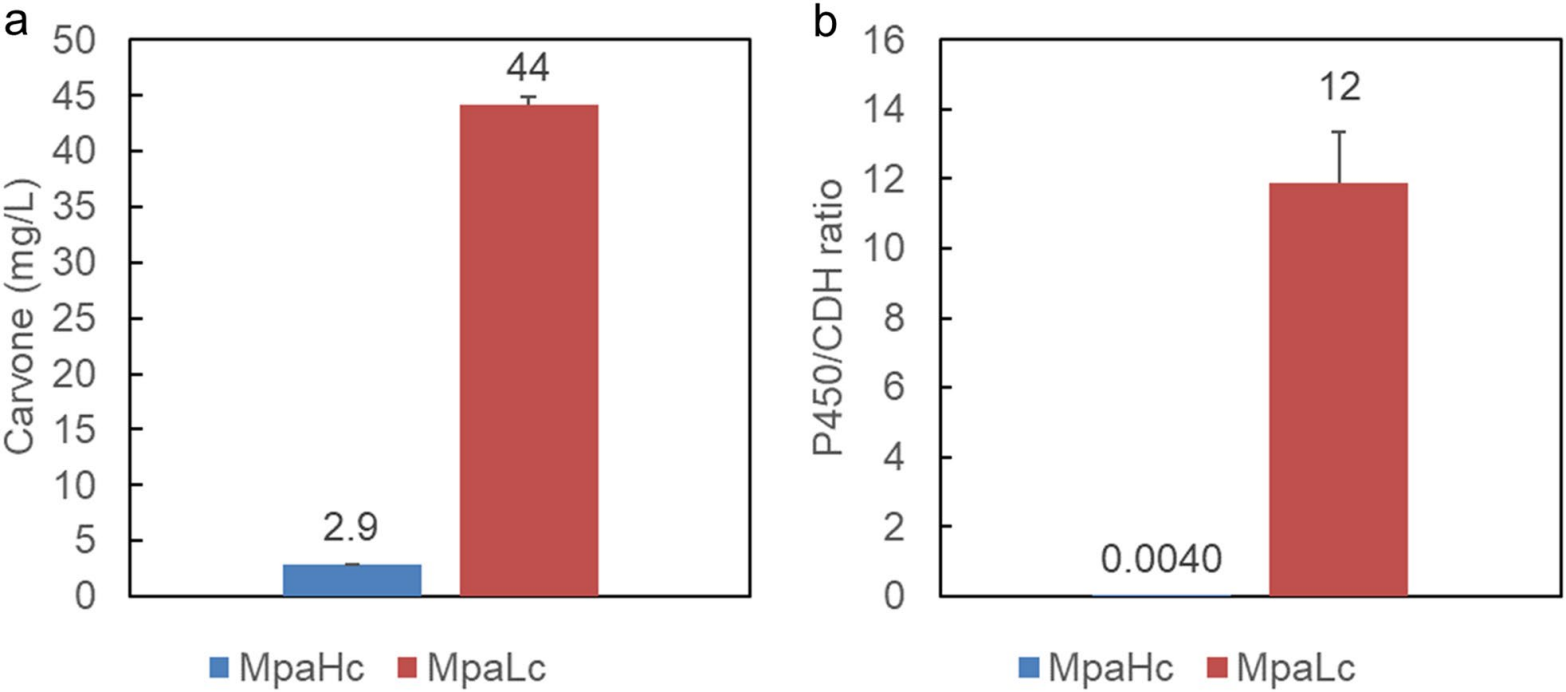
Carvone conversion via a single strain reaction. (**a**) The novel strain MpaLc (BL21(DE3) pCDF-CYP71D18-ATR2, pMW-ISPD), and the previously constructed strain MpaHc (BL21(DE3) pCDF-CYP71D18-ATR2, pET-ISPD) were analyzed using the carvone biocatalysis assay with 100 mg/L (−)-limonene as a starting substrate. Error bars represent standard deviations of *n* = 3. (**b**) The P450/CDH ratio was calculated based on quantification concatamer (QconCAT) proteome analysis of MpaHc and MpaLc strains. Error bars represent standard deviations of *n* = 3.

From the QconCAT proteome analysis results, an additional strain was constructed to represent an improved balance of P450 and CDH expression in a single strain. As the CDH gene was previously expressed from a high-copy vector pET-3a (copy number ~ 40), the low-copy vector pMW218 (copy number ~ 5) was selected as a new backbone to express CDH at decreased expression levels. The constructed plasmid pMW-ISPD was introduced into the *E. coli* BL21(DE3) strain with the P450 expression plasmid pCDF-CYP71D18-ATR2-ISPD (MpaLc). The novel strain MpaLc and previously constructed strains with pET-ISPD (MpaHc) were analyzed using the QconCAT proteome analysis and carvone biocatalysis assay. MpaLc produced 44 ± 6.3 mg/L (−)-carvone from 100 mg/L (−)-limonene as a starting substrate, whereas MpaHc produced 2.9 ± 0.79 mg/L (−)-carvone (Fig. 7). The P450/CDH ratio of MpaLc was 12 ± 1.5, whereas MpaHc was 0.004 ± 0.0008. The absolute protein abundance was shown in Supplementary Fig. S11 online, and it was shown that P450 (CYP71D18) expression was increased in the MpaLc strain. The P450/CDH ratio was higher in MpaLc as designed, and carvone biocatalysis expectedly increased.

## Discussion

Proteome data are becoming increasingly popular in metabolic engineering^22^. In this study, we successfully demonstrated the benefit of targeted QconCAT proteome analysis in genetic engineering to upgrade metabolic pathways. The QconCAT method enables quantitative analysis such that the abundance of pathway enzymes can be determined and optimized. This information is powerful when the imbalance of enzyme expression is a bottleneck in strain production. An imbalance of enzymatic pathway expression is an extremely common issue in genetic engineering, and we believe that quantitative proteome analysis would provide a clear answer in the strain development strategy. The QconCAT method requires the preparation of labeled and purified QconCAT protein, which can be a technical hurdle. In addition, the disadvantage of QconCAT was that the target protein cannot be changed after QconCAT preparation. However, novel methods are reported to overcome these issues, such as the “MEERCAT” method that combines QconCAT and cell-free expression system^23^, and the “ALACAT” method that combines QconCAT and biobricks^24^.

In this study, the labeling method was by [U-^13^C_6_] glucose. Since this method has multiple labeling sites per peptide, there are possible errors by labeling deficit. To improve the accuracy of the quantification in the future study, it is effective to use the labeling method with a single labeling site per peptide, such as arginine and lysine labeling. The quantification in this study was calculated using only the monoisotopic peak areas of labeled peptides, so that there is a potential for an error caused by a labeling deficit. Using the sum of the labeling deficit peak areas in quantification would minimize the error caused by labeling deficit for future study. In addition, the time-course trypsin digestion revealed that some peptides did not reach a plateau of digestion, thus causing an error in quantification. To improve the accuracy, it would be effective to include the recapitulate short flanking peptides, which may help to standardize the digestion rate for future studies^24^. It is important to optimize the trypsin digestion time on each QconCAT study. Excessive digestion time may cause non-specific digestion and undesired by-product, whereas insufficient digestion time may cause incomplete digestion and miscleaved peptides.

In the (−)-carvone production pathway, it was necessary to balance the expression level of P450/CPR and CDH, as excessive CDH reportedly results in by-product formation. To investigate the optimal conversion conditions, two strains independently expressing P450/CPR and CDH were mixed with different mixing ratios, and the QconCAT method subsequently revealed the optimal abundance ratio between P450/CPR and CDH. Using this proteome data, an upgraded strain was constructed to represent the optimum abundance ratio between the P450/CPR and CDH within a single strain. This upgraded strain displayed a 15-fold improvement in (−)-carvone production compared to our initial strain and achieved an increase in (−)-carvone titer approximately 150 times higher than previously reported^12^. Future studies can further improve P450/CDH ratio closer to the optimum to increase (−)-carvone production. To the best of our knowledge, this is the first report of genetic engineering to upgrade metabolic pathways using the QconCAT method.

In this study, a simple modification of CDH-expressing plasmid backbone changes to the low-copy vector dramatically increased (−)-carvone production. Carvone bioconversion pathway consists of three enzymes, so that the optimization was relatively simple. As we tackle further complicated pathways associated with additional enzymes, the optimal abundance ratio determined by the QconCAT study can serve as a design guideline to fine-tune the expression of enzymatic pathways. There are multiple techniques for fine-tuning the enzyme expression within cells, and these techniques can be combined with the optimum abundance ratio determined by the QconCAT study to successfully tweak the enzymatic pathway expression balance. The QconCAT method-based methodology that we have developed has the potential to be applied to a wide variety of target compounds in synthetic biology for fine-tuning enzyme pathway expressions.

**Figure 8:**
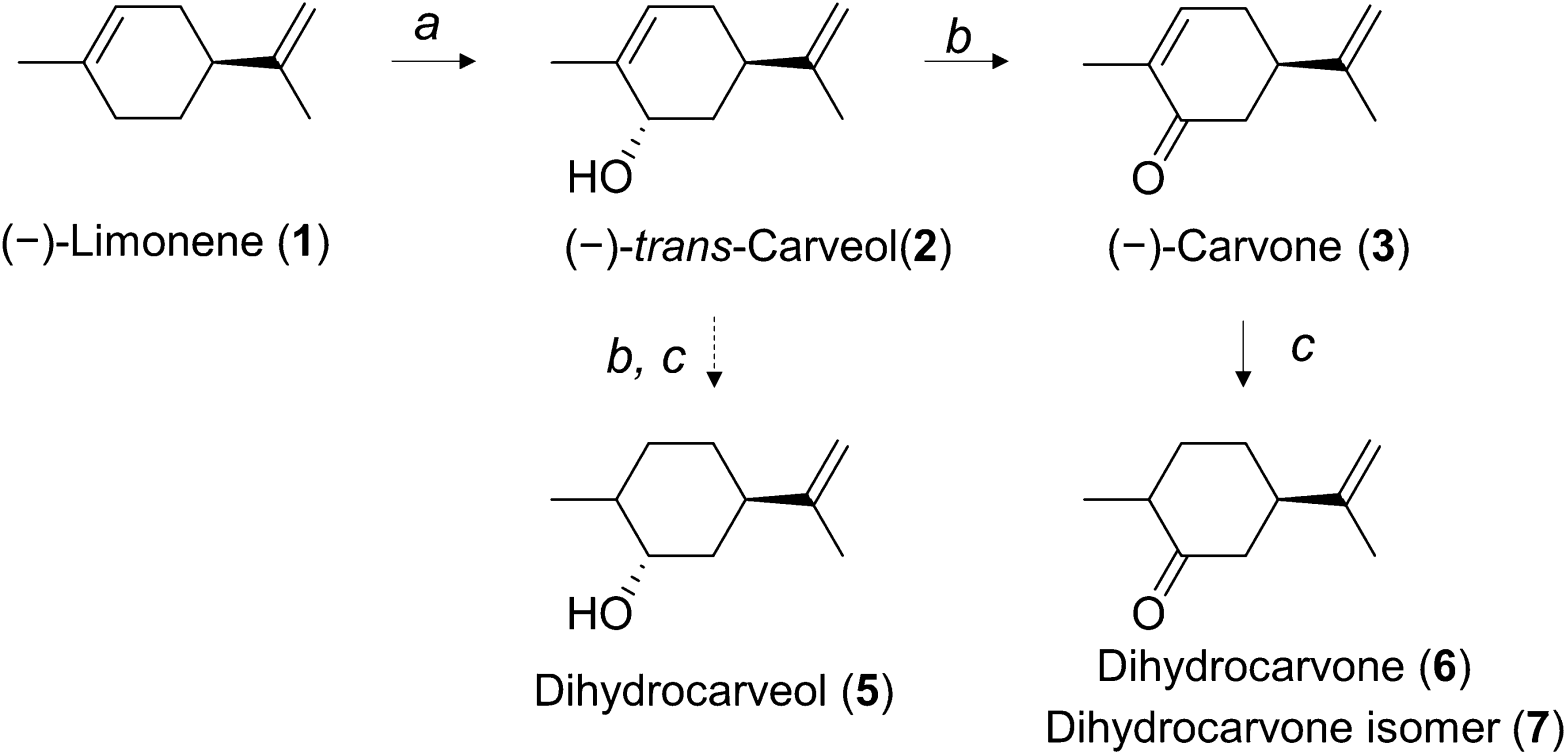
Hypothetical by-product formation pathway. Biosynthesis pathway of (−)-carvone from the primary precursor (−)-limonene. The indicated enzymes are (**a**) cytochrome P450 limonene-6-hydroxylase, (**b**) carveol dehydrogenase, and (**c**) an unknown endogenous enzyme.

For the first time, our study shows that (−)-carvone biocatalysis from (−)-limonene in engineered *E. coli* can lead to undesired by-product formation. Dihydrocarveol was detected when (−)-carveol was incubated with cells expressing CDH. Dihydrocarvone was detected when (−)-carveol was incubated with cells that did not express CDH. Dihydrocarvone was also produced when (−)-carvone was incubated with the wild type *E. coli* strain. Therefore, we propose a hypothetical by-product formation pathway (Fig. 8). ISPD, the CDH from peppermint, has been reported to be active on substrates such as (−)-*trans*-carveol, (−)-*trans*-isopiperitenol, (+)-neomenthol, and (+)-neoisomenthol^11^. Alternatively, it is not active on substrates such as (−)-*cis*-carveol, (−)-menthol, (+)-isomenthol, and (−)-perillyl alcohol^11^. ISPD was unable to catalyze the reduction of (−)-isopiperitenone or (−)-carvone^11^. There was no data concerning ISPD activity against dihydrocarveol or dihydrocarvone. Spearmint essential oil is composed of carvone (51.7%), dihydrocarveol (11.5%), and *cis*-dihydrocarvone (9.1%)^1^. However, the enzyme responsible for the formation of dihydrocarveol and dihydrocarvone in spearmint is unknown. To elucidate this pathway, an ISPD in vitro assay should be performed in future work. For industrial purposes, the reduction of by-product formation is preferred to minimize overall production costs. The *E. coli* endogenous gene responsible for the reaction from (−)-carvone to dihydrocarvone is also unknown. It is possible to search for this gene via genetic screening; however, multiple genes could be responsible for such a reaction, requiring multiple gene deletions. We undertook a realistic approach to overcome this situation by reducing the CDH expression level such that it was balanced against P450 and optimized the reaction conditions to prevent further by-product formation. In our final strain (MpaLc), dihydrocarveol and dihydrocarvone were still present. To reduce these by-products, further optimization is necessary.

The purpose of this study was to develop a method to produce natural (−)-carvone. We demonstrated *E. coli* whole-cell bioconversion from (−)-limonene as a feeding substrate. (−)-Limonene is a highly volatile compound with low water solubility. Substrate uptake can be a limitation of this reaction, as a prior study suggests^12^. Therefore, our next goal was to integrate the developed carvone conversion pathway with the limonene biosynthesis pathway. The limonene producer strain has been reported in several studies^25,26^. Thus, for future studies, we aim to construct a limonene high-producing strain and introduce the carvone conversion pathway. In such a future strain, intracellular limonene can be efficiently converted into carveol and carvone accordingly. Direct fermentation methods with high titers and productivity can reduce production costs and potentially become environmentally friendly and cost-effective methods of natural mint flavor production in the near future.

## Conclusions

The (−)-carvone biosynthesis pathway genes, P450, CPR, and CDH from plants were introduced into *E. coli* to construct the whole-cell biocatalytic system. Whole-cell biocatalysis from (−)-limonene to (−)-carvone was attempted, and by-products such as dihydrocarveol and dihydrocarvone were detected in the reaction mixture. The optimum protein expression balance was determined using the proteome analysis QconCAT method and resulted in a strain representing a superior balance between P450 and CDH, which demonstrated a 15-fold improvement compared to the non-engineered strain. The upgraded strain (MpaLc) produced 44 ± 6.3 mg/L of (−)-carvone from 100 mg/L (−)-limonene as a starting substrate, where the control strain (MpaHc) produced 2.9 ± 0.79 mg/L of (−)-carvone and attained 150 times higher production titer than the previous report. Our study showed the usefulness of the QconCAT proteome analysis method in industrial biotechnology applications. For future study, we aim to construct a limonene high-producing strain and introduce the carvone conversion pathway to realize a sustainable and cost-effective microbial replacement of (−)-carvone production.

## Methods

### Plasmid construction

The plasmids used in this study are listed in Supplementary Table S1. Codon-optimization of the *CYP71D18* gene was performed for *E. coli* using the GeneArt Strings DNA Fragments Service by Life Technologies Corporation (Carlsbad, CA, USA). Codon-optimization of the remaining genes, except *ATR2*, was performed for *E. coli* using the OptimumGene algorithm by GenScript Biotech Corporation (Piscataway, NJ, USA). Detailed sequences are shown in Supplementary Note. The hypothetical membrane-anchoring region was removed from *Arabidopsis thaliana*-derived cytochrome P450 reductase (*ATR2*, Accession number NM_119167). The DNA sample of *ATR2* was obtained by reverse transcription from *Arabidopsis thaliana* mRNA and subsequent PCR amplification. The use of plant parts in the present study complies with international, national and/or institutional guidelines. The tag sequence for soluble expression was inserted at multiple cloning site 2 (MCS2) of the pCDFDuet-1 vector to construct the pCDF-ATR2 plasmid. The hypothetical membrane-anchoring region was removed from *Mentha spicata*-derived cytochrome P450 (*CYP71D18*, Accession number AF124815) and was codon-optimized for *E. coli* and artificially synthesized. The tag sequence for soluble expression was inserted at multiple cloning site 1 (MCS1) of the pCDF-ATR2 vector to construct pCDF-CYP71D18-ATR2. The CDH (*ISPD*, Accession number AY641428) gene from *Mentha piperita* was codon-optimized for *E. coli* expression, artificially synthesized, and inserted at the SalI site (between P450 and ATR2 genes) of the pCDF-CYP71D18-ATR2 plasmid with the Shine-Dalgarno sequence to construct the pCDF-CYP71D18-ATR2-ISPD plasmid. The *ISPD* gene was also inserted at the NdeI-BamHI site of the pET-3a plasmid to construct the pET-ISPD plasmid. Similarly, *ISPD* was inserted at the KpnI-SalI site of pMW218 to construct pMW-ISPD. The CDH (*limC*, Accession number AJ006869) gene from *Rhodococcus erythropolis* DCL14 was codon-optimized for *E. coli* expression, artificially synthesized, and inserted at the NdeI-BamHI site of the pET-3a plasmid to construct the pET-limC plasmid. The QconCAT1 gene (design details are described in the QconCAT standard protein preparation) was codon-optimized for *E. coli* expression and inserted at the BamHI-XhoI site of the pET-28a plasmid to construct the pET-QconCAT1 plasmid. The QconCAT2 gene (design details are described in the QconCAT standard protein preparation) was codon-optimized for *E. coli* expression and inserted at the BamHI-XhoI site of the pET-28a plasmid to construct the pET-QconCAT2 plasmid.

### Strains

The strains used in this study are listed in Supplementary Table S2. *E. coli* BL21(DE3) was used as the host strain for protein expression and biocatalysis.

### Culture method and biocatalysis condition

The recombinant strain BL21(DE3) harboring expression plasmids was grown at 37 °C in LB medium as the seed culture. The seed culture was inoculated into 20 mL of terrific broth (TB) medium (12 g Bacto tryptone, 24 g Bacto yeast extract, 4 mL glycerol, 2.31 g KH_2_PO_4_, 12.54 g/L K_2_HPO_4_) at a ratio of 1% and incubated at 37 °C, using BR-300LF (Taitec Corporation, Saitama, Japan). For P450 expression, 80 mg/L 5-amino levulinic acid and 100 μM Fe(NH_4_)(SO_4_)_2_ were added to TB medium to facilitate heme biosynthesis^27^. The antibiotic spectinomycin (100 mg/L), carbenicillin (100 mg/L), and kanamycin (50 mg/L) were used to maintain the plasmid. All chemicals used in this study were purchased from Sigma Aldrich (St. Louis, Mo, USA), Nacalai Tesque (Kyoto, Japan), and Tokyo Chemical Industry (Tokyo, Japan). When the optical density of the culture at 600 nm reached 0.8, isopropyl β-d-1-thiogalactopyranoside (IPTG) was added at a final concentration of 50 μM and incubated at 20 °C for 16 h.

Following induction, cells were harvested by centrifugation at 20,000×*g* for 2 min at 4 °C using himac CF15RN (Eppendorf Himac Technologies, Ibaraki, Japan) and resuspended in 50 mM potassium phosphate buffer (pH 7.2) containing 5% (v/v) glycerol. The cell suspension was diluted to achieve a final concentration of OD_600_ = 20, and mixed with the substrate. Biocatalysis was performed at 14 °C for 16 h or otherwise indicated. Reactions were carried out in a gas chromatography (GC) vial or headspace vial with tightly closed lids. The vial and its contents were cooled by ice to reduce substrate volatilization, especially that of (−)-limonene.

### Analytical method

Following the conversion reaction, vials were cooled with ice, and ethyl-acetate extraction was conducted. *E. coli* BL21(DE3) with an empty plasmid were included as negative controls in these experiments. Obtained extracts were analyzed by GC. Gas chromatography-flame ionization detector (GC-FID) analysis was performed on a GC-2010Plus gas chromatograph (Shimadzu, Kyoto, Japan) equipped with an FID (at 300 °C) and DB-1 column (30 m length, 0.25 mm internal diameter, 0.25 μm film thickness, Agilent Technologies, Santa Clara, CA, USA). The analysis was carried out with a temperature program as follows: 65 °C for 5 min, 5 °C/min to 145 °C, 25 °C/min to 250 °C, and then held at 300 °C for 3 min. The carrier gas was helium (120.7 kPa, 19.6 mL/min). The injection conditions were split-flow 1:10 and 250 °C, with a linear velocity of 35.0 cm/s.

GC mass spectrometry (GC–MS) analysis was performed on a GC–MS-QP2010 system (Shimadzu) using an Rt-DEX column (30 m length, 0.25 mm internal diameter, 0.25 μm film thickness, RESTEK, Bellefonte, PA, USA). The analysis was carried out with a temperature program as follows: 50 °C for 5 min, 5 °C/min to 230 °C. The carrier gas was helium (0.7 mL/min). The injection conditions were split-flow 1:10, 220 °C, and linear velocity of 30.4 cm/s. The interface temperature was 220 °C. The detector operated in scan mode, and detection was performed in the range of m/z 40–400.

### QconCAT standard protein preparation

Two tryptic peptides were selected based on a preliminary study to represent each protein. Peptide sequences were concatenated in two different orders to produce QconCAT1 and QconCAT2 (Fig. 5A). This artificial gene was synthesized and cloned into the BamHI-XhoI site of the pET-28a expression vector by GenScript Biotech Corporation (Piscataway, NJ, USA). Internal BamHI and XhoI sites were removed by substitution with a synonymous codon. Detailed sequences are shown in Supplementary Note. Resulting QconCAT expression plasmids were transformed into *E. coli* BL21(DE3) and maintained in LB medium containing kanamycin (50 mg/L). A single colony was inoculated into 2 mL ^13^C-M9 medium (6.78 g Na_2_HPO_4_, 3 g KH_2_PO_4_, 1 g NH_4_Cl, 0.5 g NaCl, 0.24 g MgSO_4_, 11 mg CaCl_2_, 10 mg thiamine, and 10 g/1 L [U-^13^C_6_] glucose) and incubated overnight at 37 °C. This seed culture was inoculated into 40 mL ^13^C-M9 medium at a ratio of 1% and incubated at 37 °C. When the optical density of the culture at 600 nm reached 0.6, IPTG was added at a final concentration of 1 mM and incubated at 30 °C for 46 h. Cells were harvested by centrifugation at 3500×*g* for 10 min at 4 °C and resuspended in 10 mL of xTractor Buffer (Clontech Laboratories, Inc., Mountain View, CA, USA). The crude lysate was obtained according to the manufacturer’s protocol for extracting proteins from bacterial cell culture. The lysate was His-tag purified using the Capturem Maxiprep Kit (Clontech Laboratories, Inc.) according to the manufacturer’s protocol. The purified protein concentration was measured using the Bradford method. Sample purity was confirmed using sodium dodecyl sulfate–polyacrylamide gel electrophoresis.

### Proteome analysis

Total protein was extracted as described previously with minor modifications^28^. *E. coli* cells were harvested by centrifugation at 3500×*g* for 10 min at 4 °C such that the OD_600_ × volume (mL) = 50, washed once with M9 medium, and subsequently frozen at − 80 °C until analysis. Cell pellets were resuspended in 1 mL lysis buffer (50 mM 4-(2-hydroxyethyl)-1-piperazineethanesulfonic acid (HEPES) at pH 7.5, 5% (v/v) glycerol, 15 mM dithiothreitol, 100 mM KCl, and 5 mM EDTA). Resuspended cells were disrupted using a multi-bead shocker (Yasui Kikai Corporation, Osaka, Japan) with glass beads YGB01 (diameter 0.1 mm, Yasui Kikai Corporation) at 10 cycles of 2500 rpm for 30 s at 30 s intervals, and subsequently centrifuged at 3500×*g* for 10 min at 4 °C to collect the supernatant. The supernatants were used for protein quantitation via the Bradford method.

Thereafter, 50 μg of total protein and 2 μg (100.9 pmol) of QconCAT protein were supplemented with denaturing buffer (500 mM Tris–HCl at pH 8.5, 10 mM EDTA, 7 M guanidine HCl) to a total volume of 220 μL. One microliter of 50 mg/mL dithiothreitol was added and mixed by vortexing using vortex-genie 2 Mixer (Scientific Industries, Bohemia, NY, USA) at 25 °C for 1 h. Protein was subsequently alkylated with 2.5 mL of 50 mg/mL iodoacetamide (IAA) by vortexing in the dark at 25 °C for 1 h. Next, 600 μL of ice-cold methanol, 150 μL of chloroform, and 450 μL of cold water were consecutively added to lysates and gently mixed. Following centrifugation at 20,000×*g* for 5 min at 4 °C, the upper phase was discarded. Subsequently, 450 μL of methanol was added to the bottom phase and the interphase. Proteins were precipitated by centrifugation under the same conditions. Trypsin/Lys-C digestion was performed as described previously^29^. Proteins were dissolved in 9 μL of 6 M urea for 10 min by vortexing. Thereafter, 36 μL of 0.1 M Tris–HCl (pH 8.5) was added to the protein solution and mixed via sonication using Bransonic 3510 J-DTH (Emerson Japan, Kanagawa, Japan). Proteolytic digestion into peptides was performed using 1 μL of 0.5 mg/mL lysyl endopeptidase (Lys-C; Wako Pure Chemical Industries, Osaka, Japan) at a final concentration of 1% (w/w) Lys-C per sample protein and 2.5 μL of 1% w/v ProteaseMax Surfactant Trypsin Enhancer (Promega, Madison, WI, USA) at 25 °C for 3 h, followed by 1 μL of 0.5 mg/mL l-1-tosylamide-2-phenylethyl chloromethyl ketone (TPCK)-trypsin (Promega) at a final concentration of 1% (w/w) trypsin per sample protein at 37 °C for 16 h. Following trypsin digestion, 7.5 μL water and 3 mL of 50% (v/v) formic acid were added to the protein sample, which was subsequently centrifuged at 20,000×*g* for 5 min. Finally, 12 μL of the sample was mixed with 36 μL of 5% formic acid, and the mixtures were desalted using C18-StageTips^30–32^ or MonoSpin C18 column (GL sciences) with acetonitrile as wash solution and 0.1% formic acid, 95% acetonitrile as elution solution.

Samples were analyzed by nano-liquid chromatography-mass spectrometry (nano-LC–MS/MS). The nano-LC–MS/MS system comprised an LC-20Adnano and an LC–MS-8060 triple-quadrupole mass spectrometer with an electrospray ionization ion source (Shimadzu). Sample separation was performed using nano-LC (LC-20Adnano), and electrospray ionization was performed using LC–MS-8060. All analytical methods were performed as described previously^33–35^. The multiple reaction monitoring (MRM) method used to quantify five proteins was created using the open software Skyline version 4.1^36^, and is shown in Supplementary Table S3 online. Peptides were quantified by the peak area ratio of the ^12^C sample to the ^13^C sample derived from the QconCAT1 protein. Absolute quantification values were calculated using the known concentration of internal stable labeled QconCAT1 and number of unlabeled cells. The method for data-dependent acquisition mode LC–MS/MS analysis was shown in supplementary method.

## Supplementary Information

Supplementary Information 1.

Supplementary Information 2.

## Data Availability

All raw data files are deposited to jPOST (http://jpostdb.org/, ID: JPST001296 and PXD028077). Strains examined are available from the corresponding author.
