# Supplementary material for: Increased carvone production in Escherichia coli by balancing limonene conversion enzyme expression via targeted quantification concatamer proteome analysis
Source: Sci Rep. 2021 Nov 11;11:22126. doi: 10.1038/s41598-021-01469-y (PMC8586248; doi:10.1038/s41598-021-01469-y)
Supplement: Supplementary file 1 — Supplementary Information 1. [file 41598_2021_1469_MOESM1_ESM.docx]

**Supplementary Information**

**Increased carvone production in *Escherichia coli* by balancing limonene conversion enzyme expression via targeted quantification concatamer proteome analysis**

Erika Yoshida^1,2, *^, Motoki Kojima^3^, Munenori Suzuki^3^, Fumio Matsuda^4, *^, Kazutaka Shimbo^2^, Akiko Onuki^2^, Yousuke Nishio^2^, Yoshihiro Usuda^2^, Akihiko Kondo^1,5,6^, Jun Ishii^1,5, *^

1. Graduate School of Science, Technology and Innovation, Kobe University, 1-1 Rokkodai, Nada, Kobe 657-8501, Japan

2. Research Institute for Bioscience Products & Fine Chemicals. Ajinomoto Co., Inc. 1-1 Suzuki-cho, Kawasaki-ku, Kawasaki-shi, Kanagawa 210-8681, Japan

3. KNC Bio Research Center, KNC Laboratories Co., Ltd. 1-1-1, Murotani, Nishi-ku, Kobe 651-2241, Japan

4. Department of Bioinformatic Engineering, Graduate School of Information Science and Technology, Osaka University, 1-5 Yamadaoka, Suita, Osaka 565-0871, Japan

5. Engineering Biology Research Center, Kobe University, 1-1 Rokkodai, Nada, Kobe 657-8501, Japan

6. Department of Chemical Science and Engineering, Graduate School of Engineering, Kobe University, 1-1 Rokkodai, Nada, Kobe 657-8501, Japan

*****Corresponding author.

*E-mail address*: [erika.yoshida.ti4@asv.ajinomoto.com](mailto:erika.yoshida.ti4@asv.ajinomoto.com) (Erika Yoshida)

*E-mail address*: [fmatsuda@bio.eng.osaka-u.ac.jp](mailto:fmatsuda@bio.eng.osaka-u.ac.jp) (Fumio Matsuda)

*E-mail address*: [junjun@port.kobe-u.ac.jp](mailto:junjun@port.kobe-u.ac.jp) (Jun Ishii)


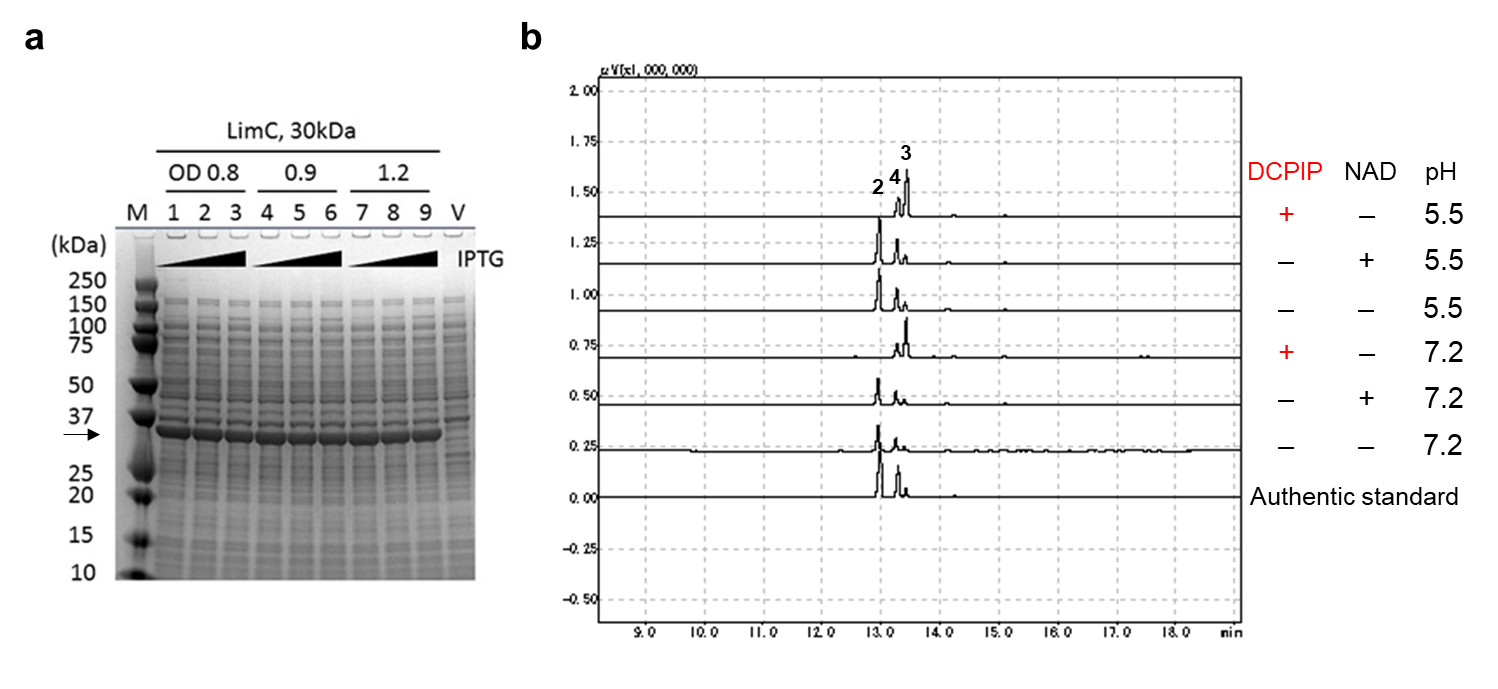


Supplementary Figure S1. Expression of *Rhodococcus erythropolis* carveol dehydrogenase in *Escherichia coli*

Carveol dehydrogenase (CDH) gene *limC* from *Rhodococcus erhthropolis* DCL14 was cloned into pET-3a and expressed in *E. coli* BL21(DE3). (**a**) Sodium dodecyl sulfate–polyacrylamide gel electrophoresis (SDS-PAGE) image showing LimC (30kDa, indicated by an arrow) was expressed in soluble fraction. Cells were induced at optical density at 600 nm as indicated. Isopropyl β-D-1-thiogalactopyranoside (IPTG) was added at final concentration of 10 μM (lane 1, 4, 7), 100 μM (lane 2, 5, 8), and 1000 μM (lane 3, 6, 9) respectively. V indicates vector control strain. M indicates size marker and the positions of the molecular mass are displayed. (**b**) Gas chromatography (GC) analysis showing LimC expressed in *E. coli* converted (−)-*trans*-carveol (**2**) to (−)-carvone (**3**), only in the presence of artificial electron acceptor dichlorophenolindophenol (DCPIP). (−)-Carveol was analyzed as an authentic standard. Commercially available (−)-carveol contains (−)-*trans*-carveol (**2**), (−)-*cis*-carveol (**4**), and trace of (−)-carvone (**3**).


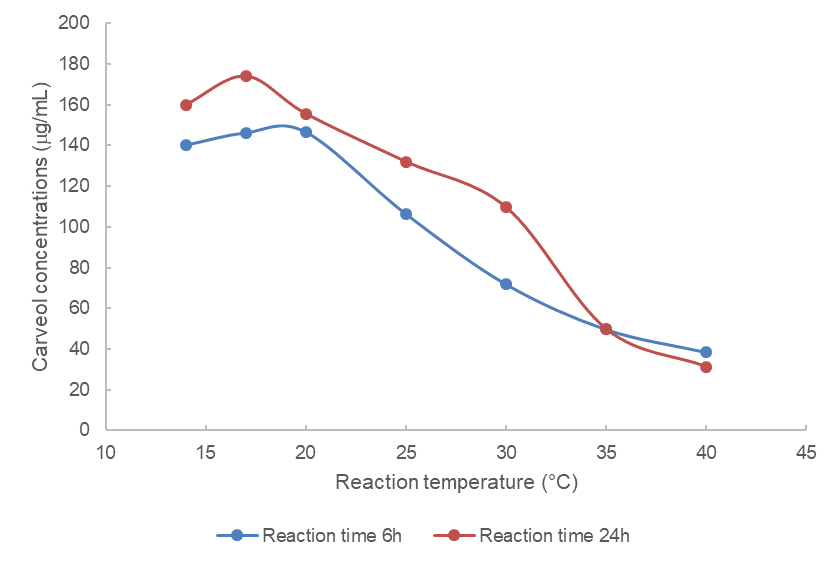


Supplementary Figure S2. Optimum reaction temperature of CYP71D18

Optimum reaction temperature of CYP71D18 was determined with (–)-limonene and cells expressing the cytochrome P450 limonene-6-hydroxylase gene from spearmint (*CYP71D18*) and cytochrome P450 reductase (CPR) gene from *Arabidopsis thaliana* (*ATR2*) (Mpa strain; BL21(DE3) pCDF-CYP71D18-ATR2)*.* Mpa strain converted (–)-limonene to (–)-*trans*-carveol within the tested temperature conditions from 14 to 40 °C. Reaction time was tested at 6 hours (blue line) or 24 hours (red line).


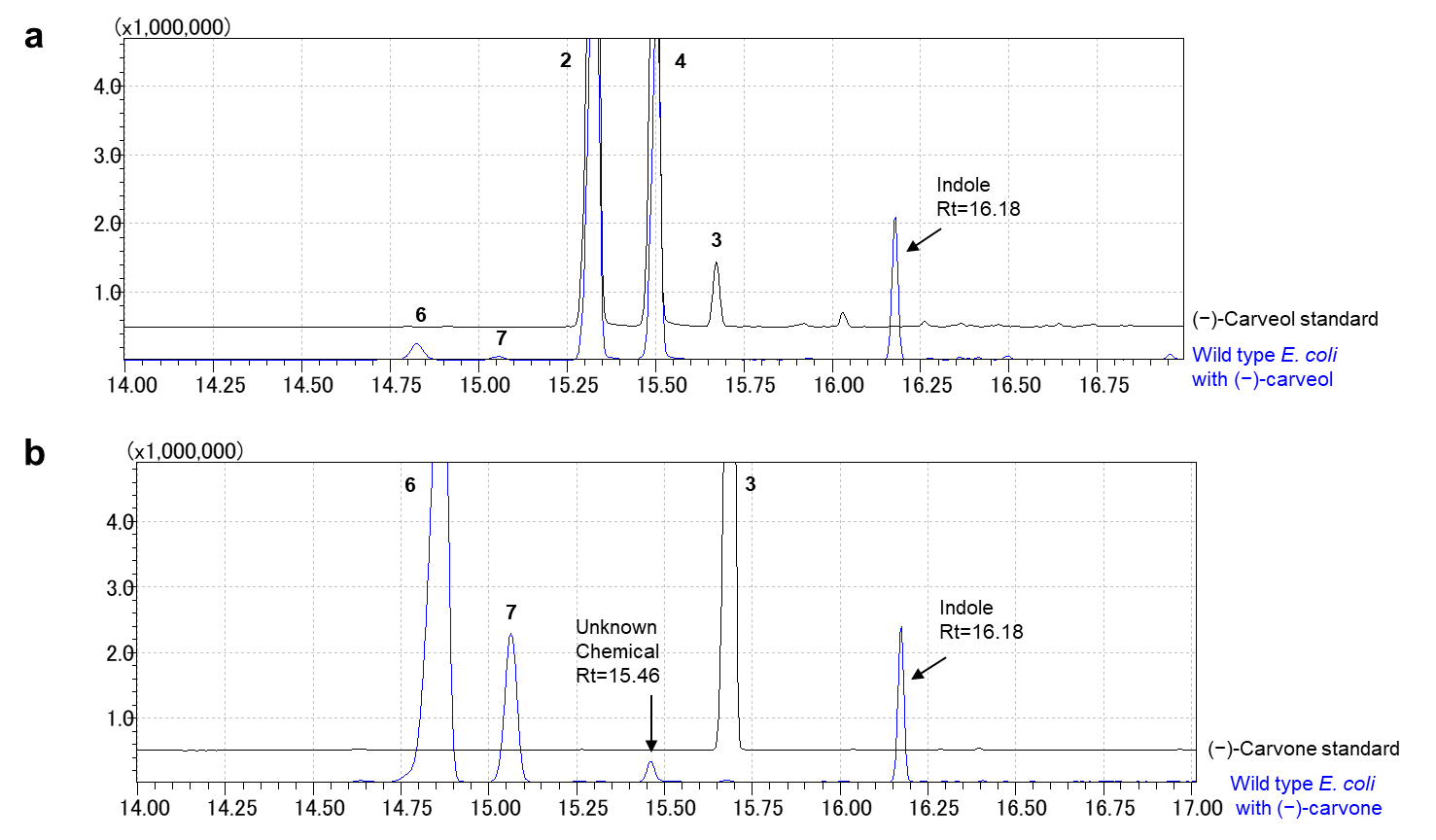


Supplementary Figure S3. By-product formation by endogenous enzyme in *Escherichia coli* BL21(DE3)

Wild type *E. coli* BL21(DE3) strain was incubated with (−)-carveol **(a)** and (−)-carvone **(b)** to determine by-product formation. Enzymatic activities were confirmed by gas chromatography (GC) analysis. Indole was observed in the mixture without substrates and is derived from *E. coli* cell solution. (**a**) Reaction with (−)-carveol and BL21(DE3) cells (blue line). The black line indicates authentic (−)-carveol standard. Commercially available (−)-carveol contains (−)-*trans*-carveol (**2**), (−)-*cis*-carveol (**4**), and trace of (−)-carvone (**3**). (**b**) Reaction with (−)-carvone and BL21(DE3) cells (blue line). The black line indicates authentic (−)-carvone standard. Numbers indicate the following compound: (−)-*trans*-carveol (**2**), (−)-carvone (**3**), (−)-*cis*-carveol (**4**), dihydrocarvone (**6**), dihydrocarvone isomer (**7**).

**
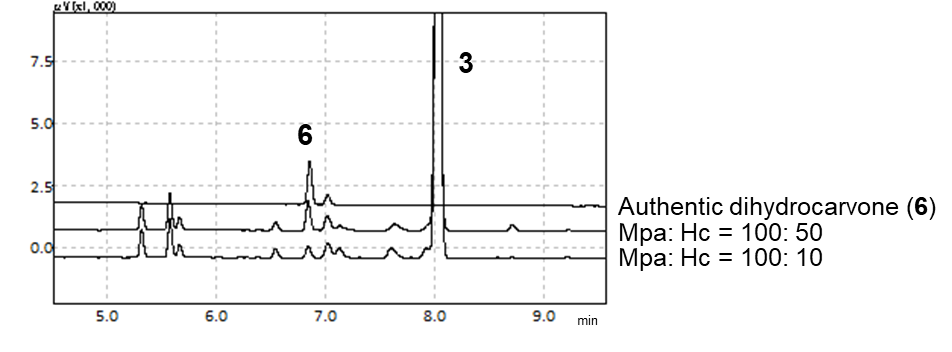
**

Supplementary Figure S4. Optimum of enzyme balance by mixing two strains expressing P450/CPR and CDH enzymes.

Strains expressing P450 and CPR (Mpa strain; BL21(DE3) pCDF-CYP71D18-ATR2) or CDH (Hc strain; BL21(DE3) pET-ISPD) were mixed in various ratios to determine the optimum balance for converting (−)-limonene to (−)-carvone. Varied P450/CDH input results in different (−)-carvone (3) production together with by-product dihydrocarvone (6) production.

**
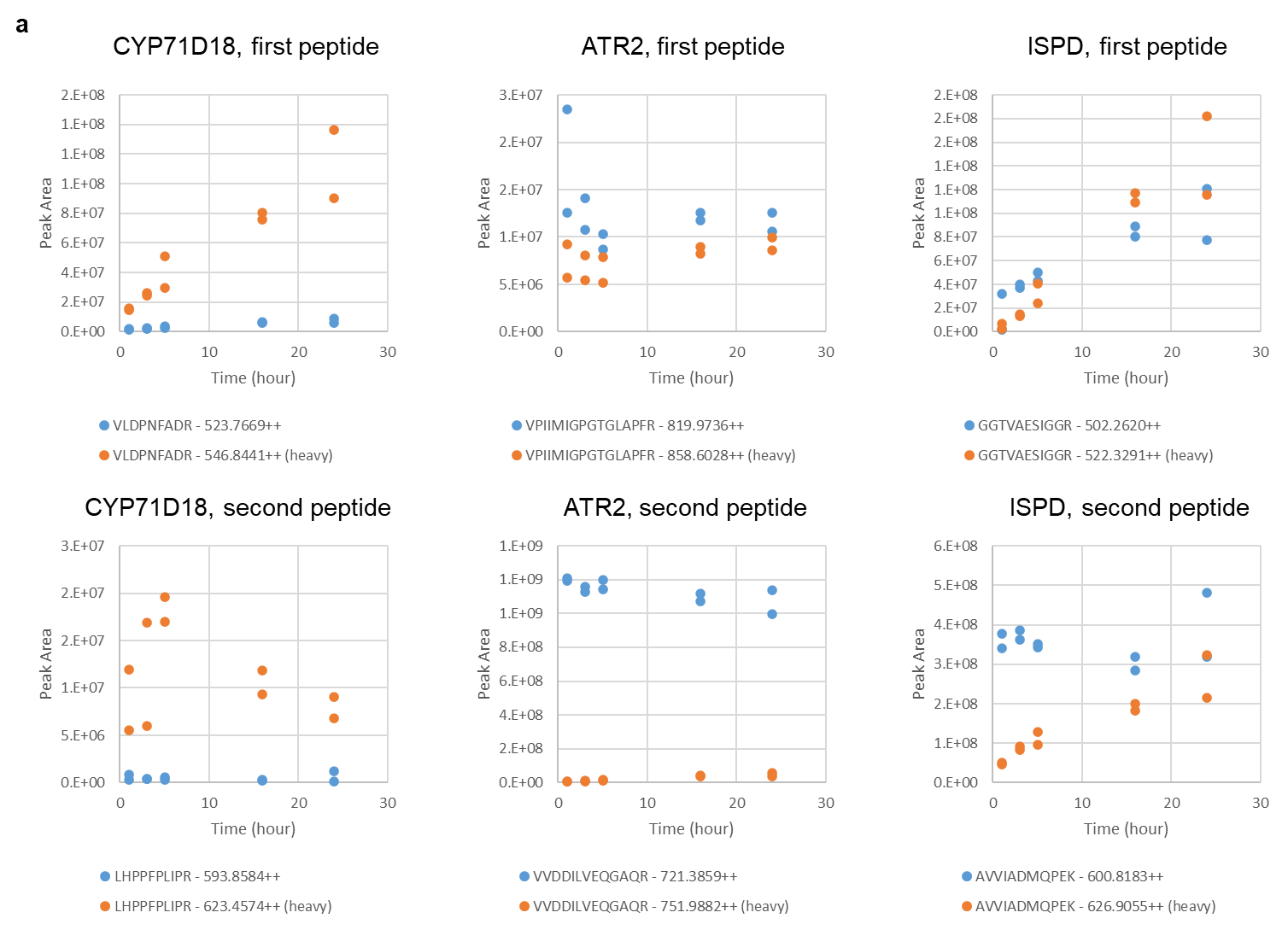
**

**
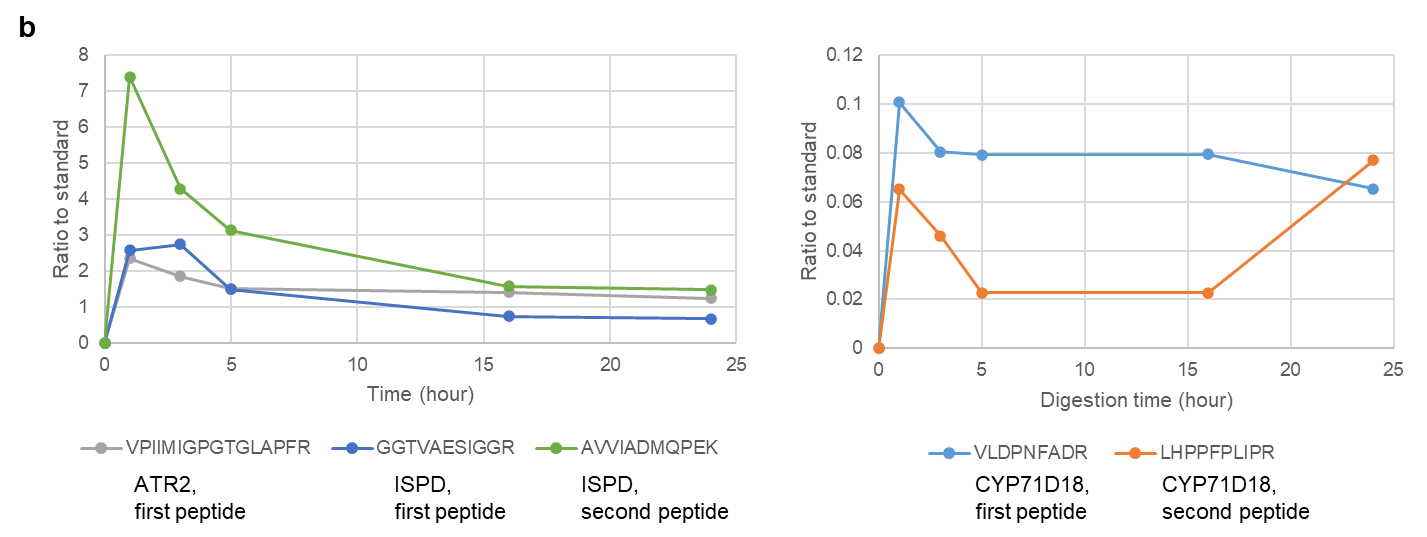
**

**Supplementary Figure S5. Time course digestion of QconCAT and sample.**

Unlabeled lysate sample (light) and labeled QconCAT1 standard (heavy) was co-digested with trypsin for 0, 1, 3, 5, 16, 24 hours. (**a**) Peak area of each peptide. Blue plot indicates the unlabeled (light) peptide and orange plot indicates the labeled (heavy) peptide. (**b**) The ratio of unlabeled (light) over labeled (heavy). The second peptide of ATR2, VVDDILVEQGAQR was excluded from quantification because the miscleavage peptide VVDDILVEQGAQREDTVLGGEYPLEK was detected.

**
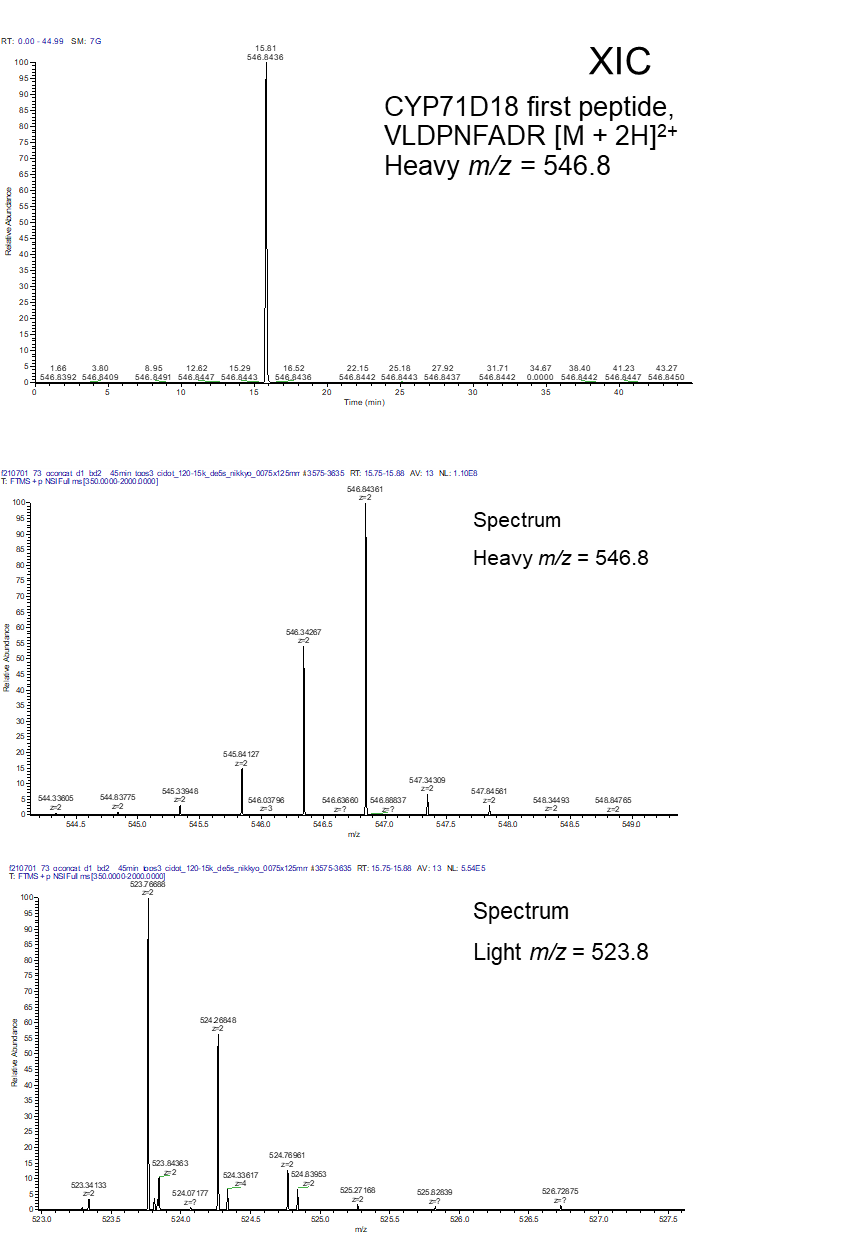
**

**Supplementary Figure S6. Isotopomer profile of labeled QconCAT.**

The extracted ion chromatogram (XIC) of labeled QconCAT peptide for CYP71D18 first peptide, VLDPNFADR [M + 2H]^2+^ and corresponding mass spectra for heavy and light labeling.

**
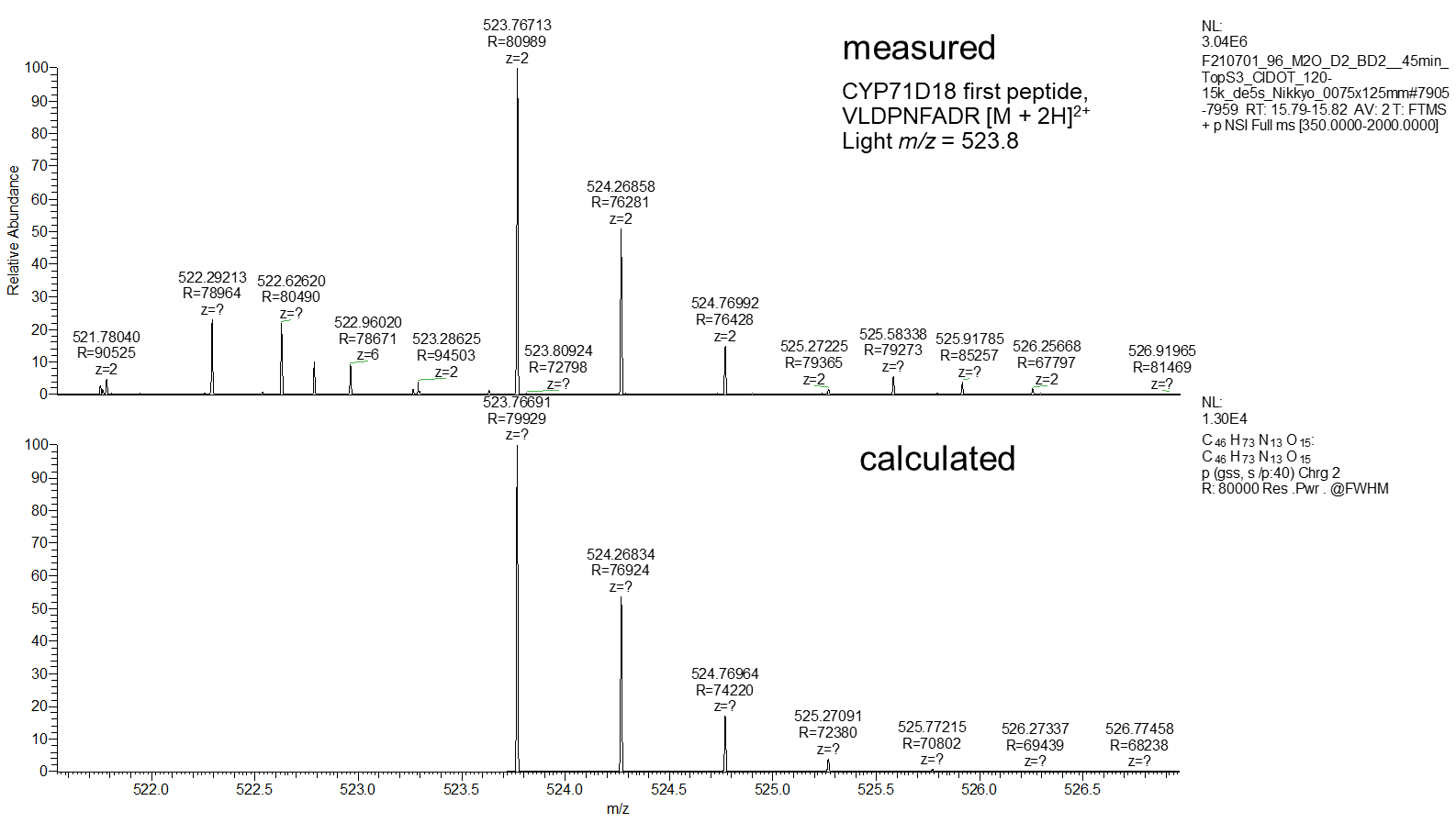
**

**Supplementary Figure S7. Isotopomer profile of unlabeled lysate** **sample.**

The measured mass spectra of unlabeled lysate sample for CYP71D18 first peptide, VLDPNFADR [M + 2H]^2+^ and calculated theoretical mass spectra.

**
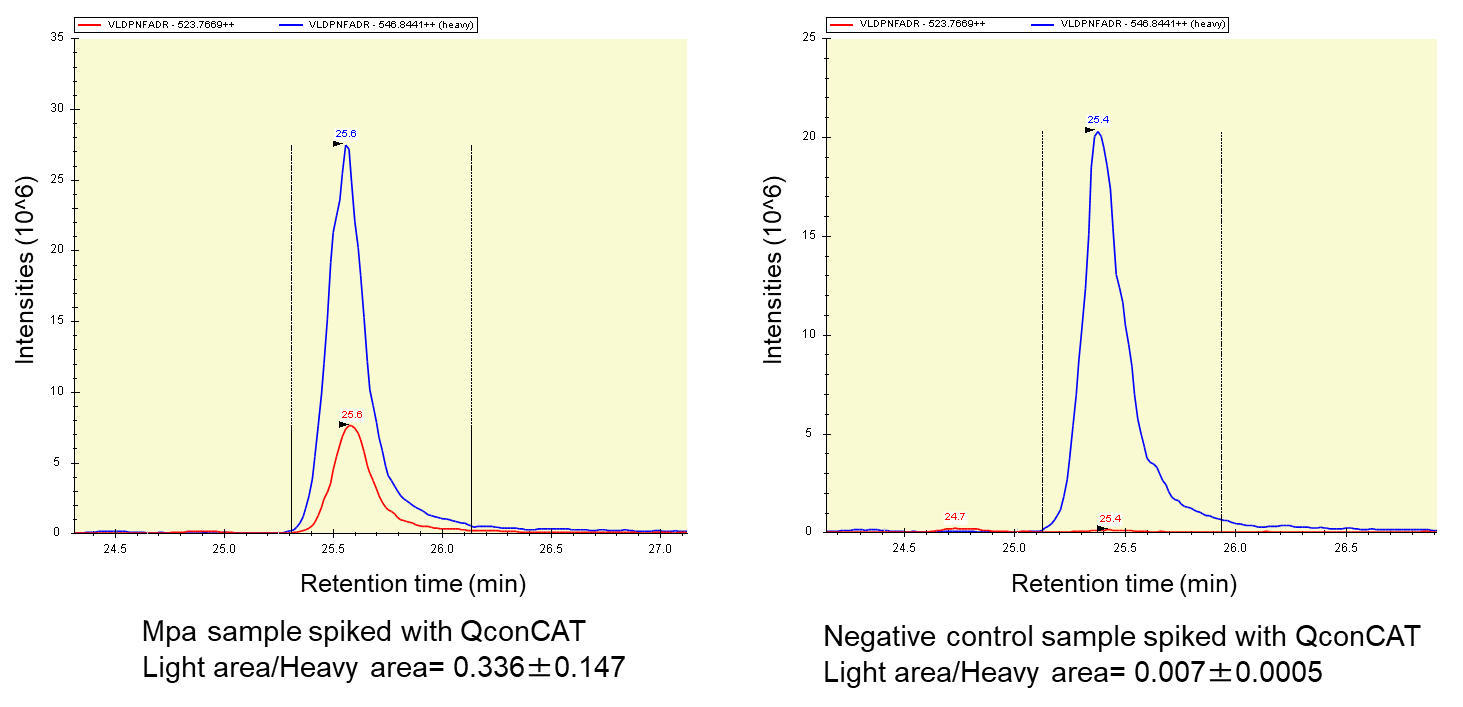
**

**Supplementary Figure S8. Effect of the labeling deficit.**

The skyline traces of CYP71D18 first peptide VLDPNFADR quantification data using Mpa strain lysate and negative control strain lysate. Mpa strain expressed P450 and CPR (BL21(DE3) pCDF-CYP71D18-ATR2) and negative control strain carried an empty vector. Both unlabeled lysate samples were spiked with labeled QconCAT1. Red line indicates intensities of light (unlabeled) VLDPNFADR peptide, and blue line indicates intensities of heavy (labeled) VLDPNFADR peptide. The effect of the labeling deficit of QconCAT1 and background is shown as the red line peak in negative control lysate.

**
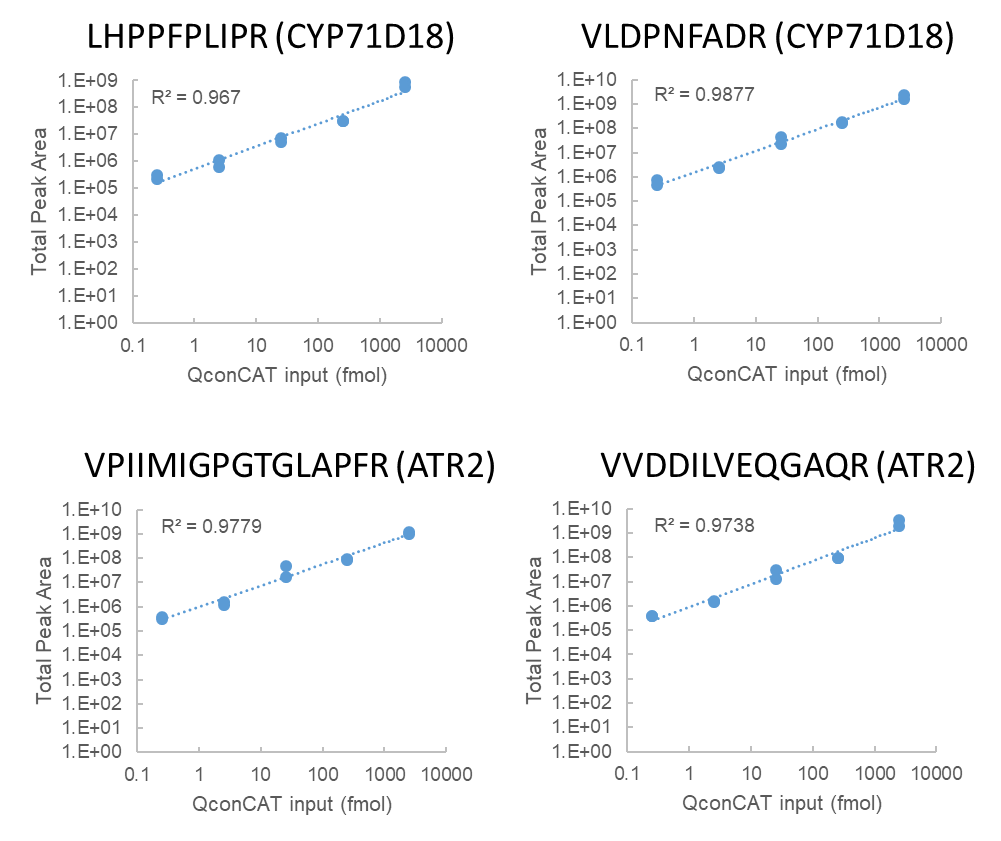
**

**
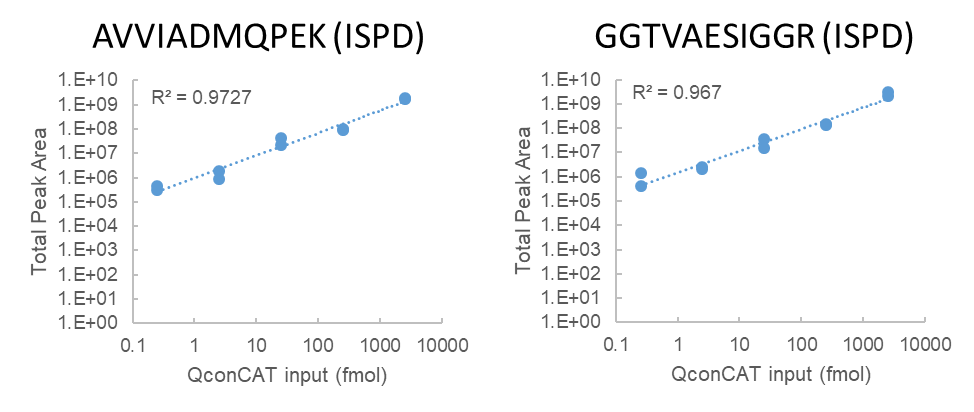
**

**Supplementary Figure S9. External calibration curve for QconCAT peptide.**

Unlabeled protein sample (light) and labeled QconCAT1 standard protein (heavy) was mixed in various ratios to make 52 μg in denaturing buffer. QconCAT protein input was 0.005, 0.05, 0.5, 5, 50 μg, which corresponds to 0.25, 2.5, 25, 250, 2500 pmol respectively.


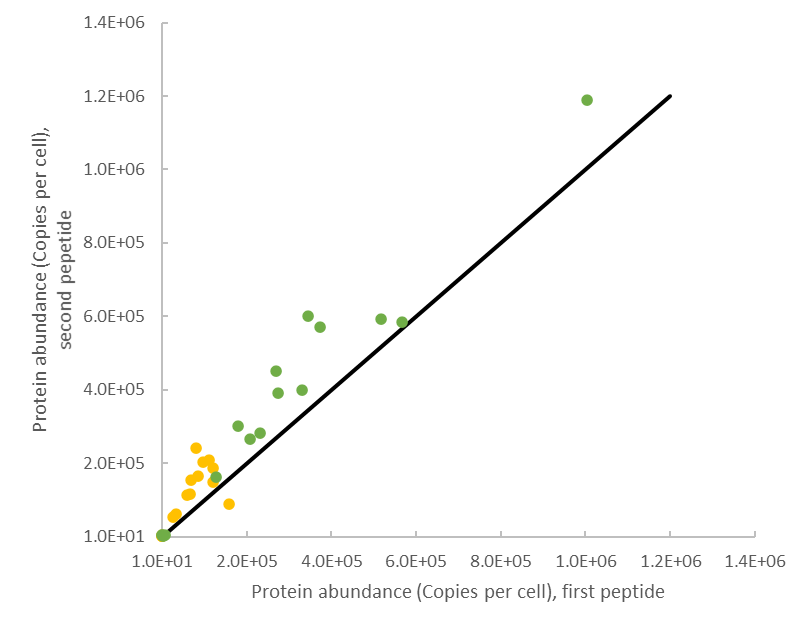


**Supplementary Figure S10. Correlation of sibling peptides.**

The correlation between values obtained with each peptide of the pair was shown. The second peptide of ATR2, VVDDILVEQGAQR was excluded from quantification because the miscleavage peptide VVDDILVEQGAQREDTVLGGEYPLEK was detected, thus the graph only displayed results of CYP71D18 (yellow plot) and ISPD (green plot). Black line indicates y=x.

**
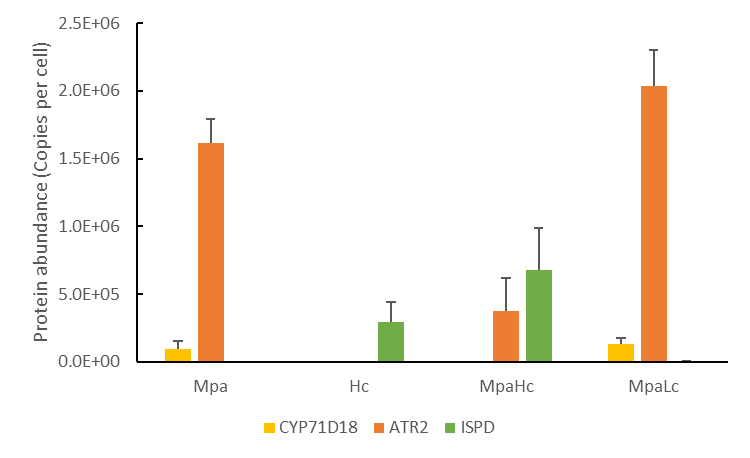
**

**Supplementary Figure S11. Absolute quantification of upgraded strain.**

Unlabeled soluble proteins from strains expressing P450 (CYP71D18), CPR (ATR2), or CDH (ISPD) were mixed with labeled QconCAT, digested with trypsin and analyzed by nano-LC-MS/MS. The intensities of unlabeled and labeled peptides were used to calculate the absolute amounts of each protein, as copies of each protein per cell. The Mpa strain (BL21(DE3) pCDF-CYP71D18-ATR2) expressed P450 (CYP71D18) and CPR (ATR2). The Hc strain (BL21(DE3) pET-ISPD) expressed CDH (ISPD). The MpaHc strain (BL21(DE3) pCDF-CYP71D18-ATR2, pET-ISPD) expressed P450 (CYP71D18), CPR (ATR2), and CDH (ISPD). The MpaLc strain (BL21(DE3) pCDF-CYP71D18-ATR2, pMW-ISPD) expressed P450 (CYP71D18), CPR (ATR2), and CDH (ISPD). The protein abundance was calculated as an average of three independent clones. Error bars represent standard deviations of n = 3.

Supplementary Table S1. Plasmids used in this study

| Plasmid name | Description | Source of reference |
| --- | --- | --- |
| pCDFDuet-1 | Expression vector carrying two multiple cloning sites with T7*lac* promoter and ribosome binding site, CloDF13-derived CDF replicon, *lacI* gene, and streptomycin/spectinomycin resistance gene. | Novagen |
| pCDF-ATR2 | Cytochrome P450 reductase (*ATR2*, Accession number NM_119167) gene derived from *Arabidopsis thaliana* was inserted at multiple cloning site 2 (MCS2) of pCDFDuet-1. | This study |
| pCDF-CYP71D18-ATR2 | Cytochrome P450 (*CYP71D18*, Accession number AF124815) gene derived from *Mentha spicata* was inserted at multiple cloning site 1 (MCS1) of pCDF-ATR2. | This study |
| pCDF-CYP71D18-ATR2-ISPD | Carveol dehydrogenase (*ISPD*, Accession number AY641428) gene from *Mentha piperita* was inserted at SalI site (in between P450 and CPR genes) of pCDF-CYP71D18-ATR2 | This study |
| pET-3a | Expression vector carrying one cloning site with T7 promoter and ribosome binding site, pBR322 replicon, and ampicillin resistance gene. | Novagen |
| pET-ISPD | Carveol dehydrogenase (*ISPD*, Accession number AY641428) gene from *Mentha piperita* was inserted at the NdeI-BamHI site of pET-3a. | This study |
| pMW218 | Cloning vector carrying one multiple cloning site, pSC101 replicon, and kanamycin resistance gene. | Nippon Gene |
| pMW-ISPD | Carveol dehydrogenase (*ISPD*, Accession number AY641428) gene from *Mentha piperita* was inserted at the KpnI-SalI site of pMW218. | This study |
| pET-limC | Carveol dehydrogenase (*limC*, Accession number AJ006869) gene from *Rhodococcus erythropolis* DCL14 was inserted at the NdeI-BamHI site of pET-3a. | This study |
| pET-28a | Expression vector carrying one cloning site with T7*lac* promoter and ribosome biding site, N-terminal His•Tag/thrombin/T7•Tag configuration, pBR322 replicon, and kanamycin resistance gene. | Novagen |
| pET-QconCAT1 | QconCAT1 sequence was inserted at the BamHI-XhoI site of pET-28a. | This study |
| pET-QconCAT2 | QconCAT2 sequence was inserted at the BamHI-XhoI site of pET-28a. | This study |

Supplementary Table S2. Strains used in this study

| Strain | Parental strain | Plasmid |
| --- | --- | --- |
| Ma | BL21(DE3) | pCDF-ATR2 |
| Mpa | BL21(DE3) | pCDF-CYP71D18-ATR2 |
| Mpac | BL21(DE3) | pCDF-CYP71D18-ATR2-ISPD |
| Hc | BL21(DE3) | pET-ISPD |
| H | BL21(DE3) | pET |
| MpaHc | BL21(DE3) | pCDF-CYP71D18-ATR2, pET-ISPD |
| Lc | BL21(DE3) | pMW-ISPD |
| MpaLc | BL21(DE3) | pCDF-CYP71D18-ATR2, pMW-ISPD |

**Supplementary Note. Detailed sequences for plasmid construction**

> pCDF-*ATR2* (Vector derived sequence of MCS2 was indicated in lower case)

cggataacaattccccatcttagtatattagttaagtataagaaggagatatacatATGACCATGATTACGAATTGGAGGAGATCCGGTTCTGGGAATTCAAAACGTGTCGAGCCTCTTAAGCCTTTGGTTATTAAGCCTCGTGAGGAAGAGATTGATGATGGGCGTAAGAAAGTTACCATCTTTTTCGGTACACAAACTGGTACTGCTGAAGGTTTTGCAAAGGCTTTAGGAGAAGAAGCTAAAGCAAGATATGAAAAGACCAGATTCAAAATCGTTGATTTGGATGATTACGCGGCTGATGATGATGAGTATGAGGAGAAATTGAAGAAAGAGGATGTGGCTTTCTTCTTCTTAGCCACATATGGAGATGGTGAGCCTACCGACAATGCAGCGAGATTCTACAAATGGTTCACCGAGGGGAATGACAGAGGAGAATGGCTTAAGAACTTGAAGTATGGAGTGTTTGGATTAGGAAACAGACAATATGAGCATTTTAATAAGGTTGCCAAAGTTGTAGATGACATTCTTGTCGAACAAGGTGCACAGCGTCTTGTACAAGTTGGTCTTGGAGATGATGACCAGTGTATTGAAGATGACTTTACCGCTTGGCGAGAAGCATTGTGGCCCGAGCTTGATACAATACTGAGGGAAGAAGGGGATACAGCTGTTGCCACACCATACACTGCAGCTGTGTTAGAATACAGAGTTTCTATTCACGACTCTGAAGATGCCAAATTCAATGATATAAACATGGCAAATGGGAATGGTTACACTGTGTTTGATGCTCAACATCCTTACAAAGCAAATGTCGCTGTTAAAAGGGAGCTTCATACTCCCGAGTCTGATCGTTCTTGTATCCATTTGGAATTTGACATTGCTGGAAGTGGACTTACGTATGAAACTGGAGATCATGTTGGTGTACTTTGTGATAACTTAAGTGAAACTGTAGATGAAGCTCTTAGATTGCTGGATATGTCACCTGATACTTATTTCTCACTTCACGCTGAAAAAGAAGACGGCACACCAATCAGCAGCTCACTGCCTCCTCCCTTCCCACCTTGCAACTTGAGAACAGCGCTTACACGATATGCATGTCTTTTGAGTTCTCCAAAGAAGTCTGCTTTAGTTGCGTTGGCTGCTCATGCATCTGATCCTACCGAAGCAGAACGATTAAAACACCTTGCTTCACCTGCTGGAAAGGATGAATATTCAAAGTGGGTAGTAGAGAGTCAAAGAAGTCTACTTGAGGTGATGGCCGAGTTTCCTTCAGCCAAGCCACCACTTGGTGTCTTCTTCGCTGGAGTTGCTCCAAGGTTGCAGCCTAGGTTCTATTCGATATCATCATCGCCCAAGATTGCTGAAACTAGAATTCACGTCACATGTGCACTGGTTTATGAGAAAATGCCAACTGGCAGGATTCATAAGGGAGTGTGTTCCACTTGGATGAAGAATGCTGTGCCTTACGAGAAGAGTGAAAACTGTTCCTCGGCGCCGATATTTGTTAGGCAATCCAACTTCAAGCTTCCTTCTGATTCTAAGGTACCGATCATCATGATCGGTCCAGGGACTGGATTAGCTCCATTCAGAGGATTCCTTCAGGAAAGACTAGCGTTGGTAGAATCTGGTGTTGAACTTGGGCCATCAGTTTTGTTCTTTGGATGCAGAAACCGTAGAATGGATTTCATCTACGAGGAAGAGCTCCAGCGATTTGTTGAGAGTGGTGCTCTCGCAGAGCTAAGTGTCGCCTTCTCTCGTGAAGGACCCACCAAAGAATACGTACAGCACAAGATGATGGACAAGGCTTCTGATATCTGGAATATGATCTCTCAAGGAGCTTATTTATATGTTTGTGGTGACGCCAAAGGCATGGCAAGAGATGTTCACAGATCTCTCCACACAATAGCTCAAGAACAGGGGTCAATGGATTCAACTAAAGCAGAGGGCTTCGTGAAGAATCTGCAAACGAGTGGAAGATATCTTAGAGATGTATGGTAAcctaggctgctgccaccgctgagcaataactagcataacccct

> pCDF-CYP71D18-*ATR2* (Vector derived sequence of MCS1 was indicated in lower case)

tggcatgcattaggatatacgactcactataggggaattgtgagcggataacaattcccctgtagaaataattttgtttaactttaataaggagatataccATGGCTCTGTTATTAGCAGTTTTTCTGAGCGCAATTATCATTCTGGTTGCCACCTATATTGTTAGCCTGCTGATTAACCAGTGGCGTAAAAGCAAAAGCCAGCAGAATCTGCCTCCGAGCCCTCCGAAACTGCCGGTTATTGGTCATCTGCATTTTCTGTGGGGTGGTCTGCCGCAGCATGTTTTTCGTAGCATTGCACAGAAATATGGTCCGGTTGCACATGTTCAGCTGGGTGAAGTTTATAGCGTTGTTCTGAGCAGCGCAGAAGCAGCAAAACAGGCAATGAAAGTTCTGGATCCGAATTTTGCAGATCGCTTTGATGGTATTGGTAGCCGTACCATGTGGTATGATAAAGACGATATTATCTTCAGCCCGTATAACGATCATTGGCGTCAGATGCGTCGTATTTGTGTTACCGAACTGCTGAGCCCGAAAAATGTTCGTAGCTTTGGTTATATTCGCCAAGAAGAAATTGAACGTCTGATTCGTCTGCTGGGTAGCAGCGGTGGTGCACCGGTTGATGTGACCGAAGAGGTTAGCAAAATGAGCTGTGTTGTTGTTTGTCGTGCAGCATTTGGTAGCGTTCTGAAAGATCAGGGTAGCCTGGCAGAACTGGTTAAAGAAAGCCTGGCACTGGCAAGCGGTTTTGAACTGGCAGATCTGTATCCGAGCAGCTGGCTGCTGAATCTGCTGTCACTGAACAAATATCGCCTGCAGCGTATGCGTCGTCGTCTGGATCATATTCTGGATGGTTTTCTGGAAGAACACCGTGAGAAAAAAAGCGGTGAATTTGGTGGTGAAGATATTGTGGATGTTCTGTTTCGTATGCAGAAAGGCAGCGATATCAAAATTCCGATTACCAGCAATTGCATCAAAGGCTTTATCTTCGATACCTTTAGTGCCGGTGCAGAAACCAGCAGCACCACCATTAGCTGGGCACTGAGCGAACTGATGCGTAATCCGGCAAAAATGGCAAAAGTTCAGGCCGAAGTTCGTGAAGCACTGAAAGGTAAAACCGTTGTTGATCTGAGCGAAGTGCAAGAACTGAAATATCTGCGTAGTGTGCTGAAAGAAACCCTGCGTCTGCATCCGCCTTTTCCGCTGATTCCGCGTCAGAGCCGTGAAGAATGTGAAGTTAATGGTTATACCATTCCTGCCAAAACCCGCATTTTTATCAATGTTTGGGCAATTGGTCGTGATCCGCAGTATTGGGAAGATCCGGATACCTTTCGTCCGGAACGTTTTGATGAAGTTAGCCGTGATTTTATGGGCAACGATTTTGAATTTATTCCGTTTGGTGCAGGTCGTCGCATTTGTCCGGGTCTGCACTTTGGTCTGGCAAATGTTGAAATTCCGCTGGCACAGCTGCTGTATCATTTTGATTGGAAACTGCCTCAGGGTATGACCGATGCAGATCTGGATATGACCGAAACACCGGGTCTGAGCGGTCCGAAAAAAAAAAATGTTTGTCTGGTTCCGACCCTGTATAAAAGCCCGTAAgatccgaattcgagctcggcgcgcctgcaggtcgacaagcttgcggccgcataatgctaaagtcgaacggaattgg

> pCDF-CYP71D18-ATR2-ISPD

ATCATTTTGATTGGAAACTGCCTCAGGGTATGACCGATGCAGATCTGGATATGACCGAAACACCGGGTCTGAGCGGTCCGAAAAAAAAAAATGTTTGTCTGGTTCCGACCCTGTATAAAAGCCCGTAAgatccgaattcgagctcggcgcgcctgcaggtcgacAGGAGGCAGCTATGGCAAGCGTGAAGAAGCTCGCAGGCAAGGTAGCCATCGTAACCGGCGGCGCCAGCGGCATCGGCGAGGTCACCGCCCGCCTCTTCGCCGAGCGCGGCGCACGCGCGGTGGTGATCGCCGACATGCAGCCCGAGAAGGGCGGTACCGTGGCGGAATCCATCGGTGGCCGGCGGTGCAGCTACGTCCACTGCGACATCACCGACGAGGAACAGGTCAGGTCCGTCGTGGATTGGACCGCCGCCACCTACGGCGGCGTCGACGTGATGTTCTGCAACGCGGGCACCGCCAGCGCCACCGCTCAGACCGTCCTGGACCTGGACCTGGCGCAGTTCGACCGCGTCATGCGCGTCAACGCCCGTGGCACGGCGGCGTGCGTGAAGCAGGCGGCGCGGAAGATGGTGGAGCTGGGGAGGGGAGGCGCTATCATCTGCACCGCCAGCGCGACGGCGAACCACGCCGGTCCCAACTTGACGGACTACATCATGTCGAAGTGCGGGGTGCTGGGGCTGGTGCGGTCGGCGAGTTTGCAGCTCGGGGTGCACGGGATTAGGGTTAACAGCGTGTCGCCGACGGCGCTGGCCACGCCGCTCACCGCGACGATCGGGCTCCGGACGGCCGCCGATGTGGAGAGCTTCTATGGGCAGGTCACGAGCTTGAAAGGGGTGGCGATCACGGCGGAGCACGTGGCGGAGGCGGTGGCGTTTCTGGCTTCGGATGAGGCGGCGTTCGTCACCGGCCATGATTTGGCTGTGGATGGTGGACTGCAGTGTTTACCATTCGTGGCCGTGGCCAAGTAAgtcgacaagcttgcggccgcataatgctaaagtcgaacggaattgg

(First capital letter section indicates partial P450 gene, lower-case letter indicates pCDFDuet-1 sequence, second capital letter with underline section indicates SD sequence, and third capital letter section indicates codon optimized ISPD sequence.)

> pET-ISPD (Vector derived sequence was indicated in lower case)

catATGGCAAGCGTGAAGAAGCTGGCAGGCAAGGTGGCAATCGTTACGGGCGGTGCGTCGGGTATCGGTGAAGTTACGGCTCGTCTGTTTGCAGAACGTGGCGCACGCGCTGTGGTTATTGCTGATATGCAGCCGGAAAAAGGCGGTACCGTGGCGGAATCAATTGGCGGTCGTCGCTGCTCGTATGTTCATTGTGATATCACGGACGAACAGCAAGTCCGTAGCGTCGTGGATTGGACCGCAGCAACGTACGGCGGTGTTGACGTCATGTTTTGCAACGCAGGTACCGCATCTGCAACCGCACAGACGGTGCTGGATCTGGACCTGGCGCAATTCGATCGTGTCATGCGCGTGAATGCGCGTGGCACGGCAGCTTGCGTCAAACAAGCGGCCCGTAAGATGGTGGAACTGGGTCGCGGCGGTGCAATTATCTGTACCGCTAGTGCGACGGTTCATCACGCTGGTCCGAACCTGACCGATTATATTATGTCCAAGTGTGGCGTGCTGGGTCTGGTTCGTAGCGCGTCTCTGCAGCTGGGCGTGCACGGTATTCGCGTTAATAGTGTCTCCCCGACCGCACTGGCAACGCCGCTGACCGCAACGATCGGTCTGCGTACCGCAGCTGATGTTGAAAGCTTTTACGGCCAAGTTACCTCTCTGAAAGGTGTCGCGATCACGGCCGAACATGTCGCTGAAGCGGTGGCCTTTCTGGCAAGCGACGAAGCGGCCTTCGTGACCGGTCACGATCTGGCAGTTGACGGTGGTCTGCAGTGCCTGCCGTTCGTGGCAGTTGCTAAGTAAggatcc

> pMW-ISPD (Vector derived sequence was indicated in lower case)

ggtaccGAAGGAGGGAATGTGATGGCAAGCGTGAAGAAGCTGGCAGGCAAGGTGGCAATCGTTACGGGCGGTGCGTCGGGTATCGGTGAAGTTACGGCTCGTCTGTTTGCAGAACGTGGCGCACGCGCTGTGGTTATTGCTGATATGCAGCCGGAAAAAGGCGGTACCGTGGCGGAATCAATTGGCGGTCGTCGCTGCTCGTATGTTCATTGTGATATCACGGACGAACAGCAAGTCCGTAGCGTCGTGGATTGGACCGCAGCAACGTACGGCGGTGTTGACGTCATGTTTTGCAACGCAGGTACCGCATCTGCAACCGCACAGACGGTGCTGGATCTGGACCTGGCGCAATTCGATCGTGTCATGCGCGTGAATGCGCGTGGCACGGCAGCTTGCGTCAAACAAGCGGCCCGTAAGATGGTGGAACTGGGTCGCGGCGGTGCAATTATCTGTACCGCTAGTGCGACGGTTCATCACGCTGGTCCGAACCTGACCGATTATATTATGTCCAAGTGTGGCGTGCTGGGTCTGGTTCGTAGCGCGTCTCTGCAGCTGGGCGTGCACGGTATTCGCGTTAATAGTGTCTCCCCGACCGCACTGGCAACGCCGCTGACCGCAACGATCGGTCTGCGTACCGCAGCTGATGTTGAAAGCTTTTACGGCCAAGTTACCTCTCTGAAAGGTGTCGCGATCACGGCCGAACATGTCGCTGAAGCGGTGGCCTTTCTGGCAAGCGACGAAGCGGCCTTCGTGACCGGTCACGATCTGGCAGTTGACGGTGGTCTGCAGTGCCTGCCGTTCGTGGCAGTTGCTAAGTAAgtcgac

> pET-limC (Vector derived sequence was indicated in lower case)

catATGGCTCGTGTGGAAGGTCAAGTGGCTCTGATTACCGGTGCCGCCCGTGGTCAAGGTCGTAGTCATGCGATTAAACTGGCCGAAGAAGGCGCGGATGTGATTCTGGTGGATGTTCCGAATGATGTGGTTGATATCGGCTATCCGCTGGGTACCGCGGATGAACTGGATCAGACCGCCAAAGATGTTGAAAACCTGGGTCGTAAAGCGATTGTGATCCATGCCGATGTTCGCGATCTGGAAAGCCTGACCGCGGAAGTGGATCGTGCCGTTAGCACCCTGGGCCGCCTGGATATTGTGAGCGCGAATGCCGGTATCGCCAGCGTTCCGTTTCTGAGCCATGATATTCCGGATAACACCTGGCGTCAGATGATTGATATCAATCTGACCGGCGTGTGGCATACCGCGAAAGTGGCCGTTCCGCATATTCTGGCGGGTGAACGCGGCGGTAGCATTGTTCTGACCAGCAGCGCCGCGGGCCTGAAAGGTTATGCCCAGATCAGCCATTATAGCGCGGCCAAACATGGCGTGGTTGGTCTGATGCGTAGCCTGGCCCTGGAACTGGCCCCGCATCGTGTGCGCGTTAACAGCCTGCATCCGACCCAGGTGAACACCCCGATGATTCAGAATGAAGGCACCTATCGTATCTTTAGCCCGGATCTGGAAAACCCGACCCGCGAAGATTTTGAAATTGCGAGCACCACCACCAATGCCCTGCCGATCCCGTGGGTGGAAAGCGTGGATGTTAGCAACGCGCTGCTGTTTCTGGTTAGCGAAGACGCACGCTACATTACGGGCGCGGCGATTCCGGTTGATGCAGGCACGACGCTGAAATAAggatcc

> pET-QconCAT1 (Vector derived sequence was indicated in lower case)

ggatccCGTGCGAATCCGGACGACCCGGCGTATGACGAAAACAAAGTTCCGGCGTTTATTGACGAGACCCTGGCGGCGAAGGTTCTGGACCCGAACTTCGCGGACCGTTTTGGCCTGGATGCGAGCCAGCAAATCCGTCTGCACCCGCCGTTCCCGCTGATCCCGCGTGTGCCGATCATTATGATTGGTCCGGGTACCGGTCTGGCGCCGTTTCGTGGTGGCACCGTGGCGGAGAGCATTGGTGGCCGTGACCTGGAGAGCCTGACCGCGGAAGTTGACCGTGCGGTGGTTATCGCGGATATGCAGCCGGAAAAGGTGGTTGACGATATTCTGGTGGAGCAGGGCGCGCAACGTGAAGATACCGTTCTGGGTGGTGAATACCCGCTGGAAAAGGTGGAAGGTCAAGTGGCGCTGATTACCGGTGCGGCGCGTctcgag

> pET-QconCAT2 (Vector derived sequence was indicated in lower case)

ggatccCGTGTGCCGGCGTTTATTGATGAGACCCTGGCGGCGAAGGACCTGGAGAGCCTGACCGCGGAAGTGGACCGTGCGAACCCGGACGATCCGGCGTACGATGAGAACAAGGCGGTGGTTATCGCGGACATGCAGCCGGAGAAAGTGGTTGACGATATTCTGGTGGAACAAGGTGCGCAGCGTCTGCACCCGCCGTTCCCGCTGATCCCGCGTGTGGAAGGTCAAGTTGCGCTGATTACCGGTGCGGCGCGTGTTCTGGACCCGAACTTCGCGGATCGTTTTGGCCTGGACGCGAGCCAGCAAATCCGTGAGGATACCGTGCTGGGTGGCGAGTATCCGCTGGAAAAGGGTGGCACCGTTGCGGAAAGCATTGGTGGTCGTGTTCCGATTATTATGATTGGTCCGGGCACCGGTCTGGCGCCGTTTCGTctcgag

**Supplementary method for data-dependent acquisition mode LC-MS/MS analysis**

Samples were prepared as described in proteome analysis method in the main manuscript. Samples were analyzed by liquid chromatography-mass spectrometry (LC-MS/MS). The LC-MS/MS system comprised an EASY-nLC1000 and Orbitrap Fusion (Thermo Fisher Scientific). Sample separation was performed using NTCC-360/75-3-125 (Nikkyo Technos) and Acclaim PepMap™ 100, 75 μm x 2cm nanoVIPER, C18 3 μm 100Å (Thermo Fisher Scientific). The data was analyzed using Thermo Xcalibur 3.0.63 (Thermo Fisher Scientific).
